# Vitamin D3 improved hypoxia-induced lung injury by inhibiting the complement and coagulation cascade and autophagy pathway

**DOI:** 10.1186/s12890-023-02784-y

**Published:** 2024-01-02

**Authors:** Chongyang Dai, Xue Lin, Yinglian Qi, Yaxuan Wang, Zhongkui Lv, Fubang Zhao, Zhangchang Deng, Xiaokai Feng, Tongzuo Zhang, Xiaoyan Pu

**Affiliations:** 1 Qinghai University, Xining, Qinghai Province 810016 People’s Republic of China; 2 Northwest Institute of Plateau Biology, Chinese Academy of Sciences, Xining, Qinghai Province 810001 People’s Republic of China; 3 West China Hospital, Sichuan University, Chengdu, Sichuan Province 610000 People’s Republic of China; 4 Qinghai Normal University, Xining, Qinghai Province 810008 People’s Republic of China; 5 Department of Pulmonary and Critical Care Medicine, Beijing Chao-Yang Hospital, Capital Medical University, Beijing, 100020 People’s Republic of China; 6 Department of Respiratory and Critical Care Medicine, Qinghai Provincial People’s Hospital, Qinghai University, Xining, Qinghai Province 810007 People’s Republic of China

**Keywords:** Hypoxia-induced lung injury, Vitamin D3, Complement and coagulation cascade, Autophagy

## Abstract

**Background:**

Pulmonary metabolic dysfunction can cause lung tissue injury. There is still no ideal drug to protect against hypoxia-induced lung injury, therefore, the development of new drugs to prevent and treat hypoxia-induced lung injury is urgently needed. We aimed to explore the ameliorative effects and molecular mechanisms of vitamin D3 (VD3) on hypoxia-induced lung tissue injury.

**Methods:**

Sprague–Dawley (SD) rats were randomly divided into three groups: normoxia, hypoxia, and hypoxia + VD3. The rat model of hypoxia was established by placing the rats in a hypobaric chamber. The degree of lung injury was determined using hematoxylin and eosin (H&E) staining, lung water content, and lung permeability index. Transcriptome data were subjected to differential gene expression and pathway analyses. In vitro, type II alveolar epithelial cells were co-cultured with hepatocytes and then exposed to hypoxic conditions for 24 h. For VD3 treatment, the cells were treated with low and high concentrations of VD3.

**Results:**

Transcriptome and KEGG analyses revealed that VD3 affects the complement and coagulation cascade pathways in hypoxia-induced rats, and the genes enriched in this pathway were Fgb/Fga/LOC100910418. Hypoxia can cause increases in lung edema, inflammation, and lung permeability disruption, which are attenuated by VD3 treatment. VD3 weakened the complement and coagulation cascade in the lung and liver of hypoxia-induced rats, characterized by lower expression of fibrinogen alpha chain (Fga), fibrinogen beta chain (Fgb), protease-activated receptor 1 (PAR1), protease-activated receptor 3 (PAR3), protease-activated receptor 4 (PAR4), complement (C) 3, C3a, and C5. In addition, VD3 improved hypoxic-induced type II alveolar epithelial cell damage and inflammation by inhibiting the complement and coagulation cascades. Furthermore, VD3 inhibited hypoxia-induced autophagy in vivo and in vitro, which was abolished by the mitophagy inducer, carbonyl cyanide-m-chlorophenylhydrazone (CCCP).

**Conclusion:**

VD3 alleviated hypoxia-induced pulmonary edema by inhibiting the complement and coagulation cascades and autophagy pathways.

**Supplementary Information:**

The online version contains supplementary material available at 10.1186/s12890-023-02784-y.

## Background

Hypoxia refers to the pathological process of abnormal metabolic function and morphological structure of tissues caused by insufficient oxygen supply or oxygen use disorder, which has serious adverse effects on the body's metabolism and other normal life activities [1, 2]. The lungs are extremely susceptible to the effects of a hypoxic environment, and symptoms such as dyspnea, chest tightness, palpitations, and insufficient oxygen supply to the body occur [3]. In addition, a hypoxic environment can cause severe lung dysfunction and injuries such as high-altitude pulmonary edema (HAPE) and pulmonary hypertension [4, 5]. In addition, if the body experiences severe trauma, infection, shock, acute lung injury, or acute respiratory distress syndrome can be rapidly induced, which can be life-threatening [6]. It is considered that the occurrence of HAPE is a complex process that involves multiple factors and many genes [7]. The happening and development of HAPE are related to obvious individual and racial diversity, and it is affected by environmental and genetic factors [8]. However, drugs that prevent hypoxia-induced lung injury remain scarce [9].

HAPE is a result of hypoxic pulmonary vasoconstriction and alveolar interstitial edema, yet its genetic mechanism remains elusive. The mechanisms of hypoxia-induced lung injury mainly include pulmonary arterial hypertension and acute non-bacterial inflammation, which are activated by increased activity of sodium ion channels and genetic changes [10–12]. Genetic changes are primarily associated with hypoxia and individual susceptibility. It may be regarded as an important molecular marker for evaluating conditions [13, 14].

Blood electrolyte balance means that the concentration of various ions in the blood of the human body is maintained within a certain range and maintains a stable state [15, 16]. These ions include sodium and potassium, among others, which play an important regulatory role between the inside and outside of the cells [17]. Blood electrolyte homeostasis is crucial for maintaining human health [18]. In a normal physiological state, the disparity in concentration between extracellular and intracellular compartments ensures proper cellular function [19]. However, certain conditions, such as hypoxia following carbon dioxide inhalation, can disrupt the delicate balance of electrolytes [20]. Another study showed that long-term monitoring of blood sodium levels was associated with the progression of HAPE patients and persistent abnormal blood sodium was related to higher mortality [11].

As the most active form of VD3, 1α, 25-dihydroxyvitamin D3 (1,25(OH)_2_D_3_) exerts various physiological activities, such as calcium and phosphorus regulation, immunoregulation, anti-cancer, and cardiovascular regulation [21, 22]. Ten 1,25(OH)_2_D_3_ drugs are used for the treatment of osteoporosis, psoriasis, and hyperparathyroidism. Studies have shown that VD3 plays a protective role against lung injury [23, 24]. Treatment with VD3 ameliorated seawater aspiration-induced inflammation and pulmonary edema by inhibiting NF-κB and RhoA/Rho kinase pathway activation [25]. In the lungs of hamsters with acute lung injury, VD3 treatment alleviated lipopolysaccharide (LPS) instillation [26]. High dietary VD3 intake results in elevated serum 25D3 levels and significant inhibition of lung tumor growth [27]. Furthermore, VD3 significantly reduced the expression of TLR4, NF-κB, and the inflammatory cytokines TNF-α, IL-1β, and IL-6 [24]. However, the modulatory mechanism of VD3 in protecting alveolar epithelial cells from hypoxia-induced lung injury has not yet been investigated.

In this study, we aimed to explore the mechanism underlying the protective effect of VD3 on hypoxia-induced lung injury using transcriptomic profiling. We found that the complement and coagulation cascades are downstream signaling pathways that affect the protective effect of VD3 against plateau lung injury. Thus, VD3 may play a cytoprotective role by inhibiting mitophagy.

## Methods

### Animals

Specific pathogen-free (SPF) healthy male SD rats (6–8 weeks old and weighing 180–220 g) were purchased from Chengdu Dossy Experimental Animals Co., Ltd. (Chengdu, Sichuan). The feeding environment was 25 °C ± 1 °C, relative humidity 50%-60%, and light/darkness for 12 h circulation. The rats were allowed free access to food and water. Animals and experimental protocols were conducted according to the ARRIVE guidelines.

### Establishment and grouping of animal models

Thirty SD rats were randomly divided into normoxia, hypoxia, and VD3 (1,25(OH)_2_D_3_) groups after 3 days of acclimatization in the animal experiment department, with 10 rats in each group. Rats in the VD3 group were administered 0.03 µg/kg VD3 (C9756, Sigma‐Aldrich, Missouri, USA) dissolved in peanut oil, as previously described [28]. Rats in the normoxic and hypoxic groups were administered the same amount of peanut oil. The drug was administered continuously for seven days. From the 4th day, except for the normoxia group, the other groups were placed in the simulation chamber of a 6500 m altitude environment for 72 h in a hypobaric chamber (DYC-9070; Avic Guizhou Fenglei Aviation Armament Co., Ltd., Anshun, China) as described previously [29, 30]. After entering the experimental chamber at 8:30 a.m. every day, the chamber ascended to an altitude of 4000 m at a constant speed of 2 m/s, while the simulated chamber descended to an altitude of 4000 m at a constant speed of 10 m/s. When the two chambers were stabilized, the experimenter entered the simulated chamber, administered the drugs by gavage, and changed the food and bedding. After each drug administration, the simulated chamber was returned to an altitude of 6500 m, and the experimental chamber was returned to the local altitude. During the experiment, the animals were fed and drank freely, and their survival status was monitored.

### Arterial blood gas analysis

Arterial blood gas analysis was performed seven days after administration. The rats were anesthetized via intraperitoneal injection of pentobarbital sodium (45 mg/kg). Blood was drawn from the femoral artery via femoral artery catheterization under anesthesia. Arterial blood was measured within 15 min using an ABL800 blood gas analyzer (ABL 80 Flex Basic, Radiometer, Denmark). The indices included arterial partial pressure of carbon dioxide (PaCO_2_), arterial partial pressure of oxygen (PaO_2_), arterial oxygen saturation (SaO_2_), sodium (Na^+^), potassium (K^+^), and calcium (Ca^2+^) levels.

### Measurement of pulmonary artery pressure

The right external jugular vein was separated, the distal end ligated, the proximal end clamped with an arterial clamp, and the external jugular vein removed. After endotracheal intubation and mechanical ventilation, changes in the pressure waveform were observed using the PowerLab system (PowerLab 7.8, AD Instruments, Colorado Springs, CO). The catheter was gradually inserted into the superior vena cava, progressing into the right atrium where a minor pressure waveform was observed. Subsequently, the catheter was further advanced into the right ventricle, enabling recording of the pressure curve in the right ventricle. The catheter is then sent to the pulmonary artery under the action of the right ventricular blood flow, in addition, changes in pulmonary arterial pressure waveforms were observed using PowerLab physiological loggers (ADI, Australia), and pulmonary arterial pressure was collected through a pressure sensor.

### Lung water content

The water content of the lung tissue was detected using the dry–wet weight technique, as described previously [31]. The isolated tissues of the left upper lobe of the lung were placed in a 55 °C electric heating constant temperature air drying oven until the dry weight error was within 0.0002 g [32]. Water content = wet weight—dry weight.

### RNA extraction

Total RNA was extracted from lung tissues using the Trizol-centrifuge column method (Invitrogen, San Diego, CA, USA) according to the manufacturer's instructions. DNA concentration and purity were measured using a NanoDrop 2000 spectrophotometer ( Thermo Scientific). RNA integrity number (RIN) was evaluated using an Agilent 2100 Bioanalyzer system (Santa Clara, CA, USA).

### Library construction and sequencing

After total RNA extraction, the mRNA was enriched. Complementary DNA (cDNA) was synthesized from the fragmented RNA, followed by end repair. The ligated products were amplified by bridge PCR, using specific primers to construct cDNA libraries for sequencing. After the library was successfully constructed, the BGISEQ-500 platform of the BGI Genomics Institute (BGI-Shenzhen, China) was used for high-throughput sequencing.

### Data processing and analysis

Low-quality bases, N-bases, or low-quality reads were filtered out, and high-quality clean reads were obtained. The fragments per kilobase million (FKPM) method was used to calculate the genes. Express quantity to |log_2_ (fold change)|> 1 and a false discovery rate (FDR) < 0.001 were used as the criteria to screen differentially expressed genes (DEGs). A Pearson Correlation test was performed for statistical analysis, and the results are presented in a volcano plot. Kyoto Encyclopedia of Genes and Genomes (KEGG) pathway enrichment analyses were performed to determine the functions of DEGs. KEGG pathway enrichment statistical analysis was performed using KOBAS software.

### Hematoxylin and eosin (H&E) stain

The lung tissues of the rats were collected and fixed in 4% paraformaldehyde overnight, processed, and embedded in paraffin. The tissue sections were stained with H&E to observe the degree of lesion and inflammatory cell infiltration under a 400 × magnification optical microscope (Olympus BH2, Tokyo, Japan).

### Enzyme-linked immunosorbent assay (ELISA)

ELISA was used to detect the expression levels of Fga, Fgb, PAR1, PAR3, PAR4, tumor necrosis factor-alpha (TNF-α), interleukin 6 (IL-6), and interleukin-1beta (IL-1β) in the lung tissue according to the manufacturer’s instructions. In addition, the expression levels of tissue factor (TF), coagulation factor VII (FVII), coagulation factor II (FII), coagulation factor V (FV), coagulation factor X (FX), and thrombomodulin (TM), in liver tissue were detected by ELISA method according to the kit manufacturer’s instructions. The levels of lactate dehydrogenase (LDH), TNF-α, IL-6, and IL-1β in the cell supernatant were measured by ELISA according to the manufacturer’s instructions.

### Western blot analysis

Lung tissue lysis solution or cells were prepared using RIPA buffer (Signaling Technology, Inc.). Protein concentration was determined using a BCA kit (Sigma-Aldrich; Merck KGaA). Total protein (30 µg/sample) was separated using 10% SDS-PAGE and nitrocellulose membranes. The membranes were blocked with 5% non-fat dried milk. Subsequently, the membranes were probed with primary antibodies. The membranes were washed with Tris-buffered saline/0.1% Tween (TBST) and incubated for 1.5 h with HRP Goat anti-rabbit IgG (Abcam, ab6721). Band visualization was carried out using the ECL system (Affinity Biosciences, Cincinnati, Ohio, USA) and β-actin was used as an internal control.

### Real-time fluorescence quantitative polymerase chain reaction (RT-qPCR)

Total RNAs were isolated using TRIzol® reagent (Thermo Fisher, Massachusetts, USA). cDNA was obtained using a reverse transcription kit (Invitrogen). The relative levels of target gene RNA transcriptomes were determined by qRT-PCR using the SYBR Premix Ex Taq kit (Bao Biological Engineering, Dalian, China). The reverse transcription reaction conditions were as follows: 95 °C for 30 s, 40 cycles of 95 °C for 5 s, and 60 °C for 30 s. The relative gene expression level was determined by applying the 2^−△△Ct^ method to ABI software (Foster City, CA, USA).

### Immunohistochemistry (IHC) stain

IHC staining was performed to detect protein expression in rat lung tissue. Complement (C) C3, C3a, C5, Fga, and Fgb protein expression levels were assessed according to the IHC protocol.

### Immunofluorescence (IF) staining

After the experiments, lung tissues were dissected and fixed with 4% paraformaldehyde. Paraffin sections of the lung tissues were dewaxed and hydrated. The sections were incubated in QuickBlock Blocking Buffer (Beyotime, Shanghai, China) for 30 min at room temperature. Then, The sections were incubated with the anti-ZO1 tight junction (Abcam, ab221547; 1/100) at 4 °C overnight and washed three times with phosphate-buffered saline (PBS). Staining of the lung tissues was observed under a fluorescence microscope BX53 (Olympus, Tokyo, Japan) at 400 × magnification.

### Cell culture

Rat type II alveolar epithelial cells (CP-R003) and hepatocytes (CL-0038) were purchased from Wuhan Procell Life Technology (Wuhan, China). The cells were maintained in DMEM supplemented with 10% fetal bovine serum (FBS; Gibco).

### Cell co-culture

To investigate the regulatory role of hypoxic or/and VD3-induced alveolar epithelial cells in the ability of hepatocytes to secrete coagulation factors, we developed a co-culture system. Logarithmic growth phase rat type II alveolar epithelial cells (1.0 × 10^5^/mL) were inoculated on the base of a 6-well plate (2 mL per well). For experiment 1, cells were divided into four groups: normoxia, hypoxia, hypoxia + low-concentration VD3, and hypoxia + high-concentration VD3. For experiment 2, the cells were divided into five groups: normoxia, hypoxia, hypoxia + VD3, hypoxia + VD3 + mitochondrial autophagy inhibitor (5 μM Mdivi-1), and hypoxia + VD3 + mitochondrial autophagy agonist (50 μM CCCP). The Rat type II alveolar epithelial cells were incubated under normoxic (95% O_2_, 5% CO_2_, 37 °C) conditions for 1 h and then in hypoxia (1% O_2_, 94% N_2_, 5% CO_2_, 37 °C) for 24 h, as the previously described [33]. For VD3 treatment, rat type II alveolar epithelial cells were treated with low VD3 (20 nM) or high VD3 (40 nM) for 24 h. For the mitochondrial fission inhibitor Mdivi-1 (Beyotime, Shanghai, China), rat type II alveolar epithelial cells were treated with 5 μM Mdivi-1. For treatment with the mitochondrial uncoupler carbonyl cyanide m-chlorophenylhydrazone (CCCP, Sigma-Aldrich, Missouri, USA), rat type II alveolar epithelial cells were treated with 50 μM CCCP. Logarithmic growth phase rat hepatocytes (1.0 × 10^5^/mL) were inoculated into the upper chamber of Transwell ventricles (2 mL per well). Rat hepatocytes were cultured at 37 °C and 5% CO_2_, and Traswell was placed in a 6-well plate for co-culture after the rat hepatocytes were attached to the wall overnight.

### CCK-8 assay

The viability of type II alveolar epithelial cells was measured using the Cell Counting Kit 8 (CCK-8, Thermo Fisher Scientific) according to the manufacturer’s instructions. The absorbance was recorded at 450 nm.

### JC-1 mitochondrial membrane potential detection assay

The JC-1 assay was performed to measure mitochondrial membrane potential (ΔΨm) using the JC-1 mitochondrial membrane potential assay kit (KeyGen, China). Briefly, cells were seeded in a 6-well plate and ΔΨm was detected according to the guidelines of the JC-1 kit. All samples were analyzed using an Accuri or LSRII flow cytometer (BD Biosciences).

### Transmission electron microscopy (TEM)

Transmission electron microscopy (TEM) was performed to observe autophagosomes. Briefly, prefixed with 3% glutaraldehyde, then the cells were postfixed in 1% osmium tetroxide, dehydrated in a series of acetone, infiltrated in Epox 812 for a longer period, and embedded. The semi-thin sections were stained with methylene blue, and ultrathin sections were cut with a diamond knife and stained with uranyl acetate and lead citrate. The sections were examined using a JEM-1400-FLASH Transmission Electron Microscope (JEOL JEM-1400, JEOL, Ltd., Tokyo, Japan).

### Statistical analysis

Means and standard deviations (SD) were used to represent the data. All data were analyzed using the SPSS software (version 22.0; IBM Corp., Armonk, NY, USA). Kolmogorov–Smirnov tests revealed that the data were normally distributed, that is, variables were parametric. Statistical analyses were performed using the independent sample Student’s *t*-test for comparisons between two groups. One-way ANOVA with Tukey’s post hoc test of means was used for multiple group comparisons. Statistical significance was set at *P* < 0.05.

## Results

### VD3 attenuated lung edema

**Fig. 1:**
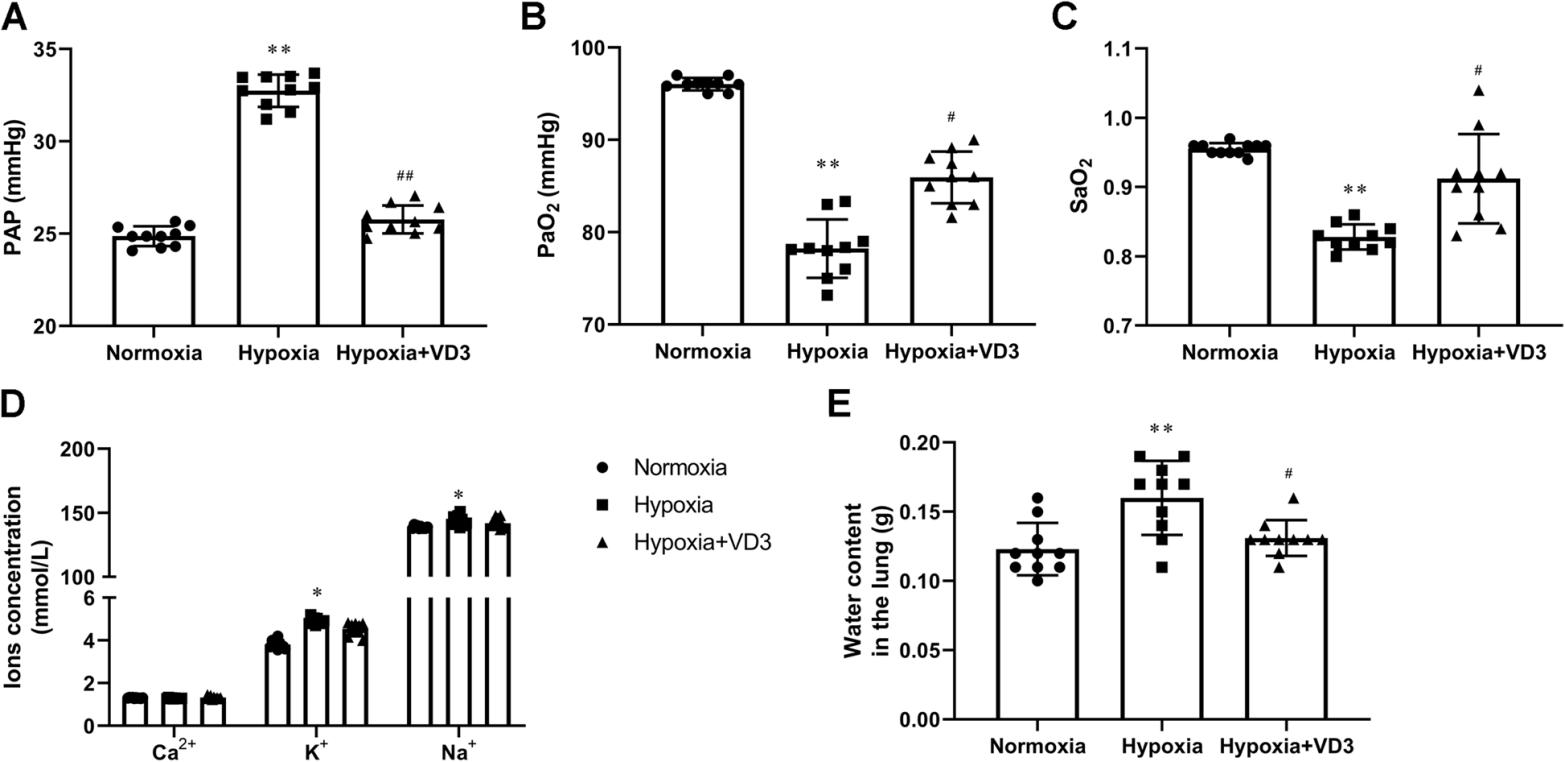
VD3 attenuated lung edema. Thirty SD rats were randomly divided into the normoxia group, hypoxia group, and vitamin D3 (VD3, 1,25-(OH)2-D3) group with 10 rats in each group. **A** Pulmonary arterial pressure was measured using a PowerLab system. **B**-**D** PAP, PaO_2_, SaO_2_, Ca^2+^, K^+^, and Na^+^ was tested using a blood gas analyzer. **E** Rat lungs were removed, and percent water content measured. Means and standard deviations (SD) were used to represent the data. Statistical analyses (two group comparisons) were performed using Students* t*-test.^*^ *P* < 0.05 vs Normoxia, ^**^ *P* < 0.01 vs Normoxia, ^#^ *P* < 0.05 vs Hypoxia, ^##^ *P* < 0.01 vs Hypoxia

First, we examined the effects of VD3 on lung edema and changes in the blood gas indices. As displayed in Fig. 1A-C, we found increased levels of pulmonary arterial pressure (PAP, 24.87 ± 0.57 vs 32.74 ± 0.93) and reduced levels of PaO_2_ (96.00 ± 1.00 vs 78.20 ± 3.11) and SaO_2_ (0.95 ± 0.01 vs 0.83 ± 0.02) after hypobaric hypoxia exposure. These changes were reversed by VD3 treatment (PAP, 32.74 ± 0.93 vs 25.78 ± 0.80; PaO_2_, 78.20 ± 3.11 vs 85.40 ± 2.88; SaO_2_, 0.83 ± 0.02 vs 0.91 ± 0.01). Moreover, the contents of sodium (138.83 ± 1.10 vs 144.20 ± 3.95), potassium (3.82 ± 0.20 vs 4.92 ± 0.15), and calcium (1.31 ± 0.02 vs 1.29 ± 0.02) ions were significantly augmented after hypobaric hypoxia exposure (Fig. 1D). VD3 treatment inhibited the occurrence of the phenomenon (sodium, 144.20 ± 3.95 vs 141.83 ± 3.97; potassium, 4.92 ± 0.15 vs 4.53 ± 0.29; calcium, 1.29 ± 0.02 vs 1.32 ± 0.07). We demonstrated that VD3 reduced the increase in lung water content after hypobaric hypoxia exposure, suggesting remission of lung edema (Fig. 1E).

### Effect of VD3 on transcriptome differential genes in a hypoxia-induced rat

**Fig. 2:**
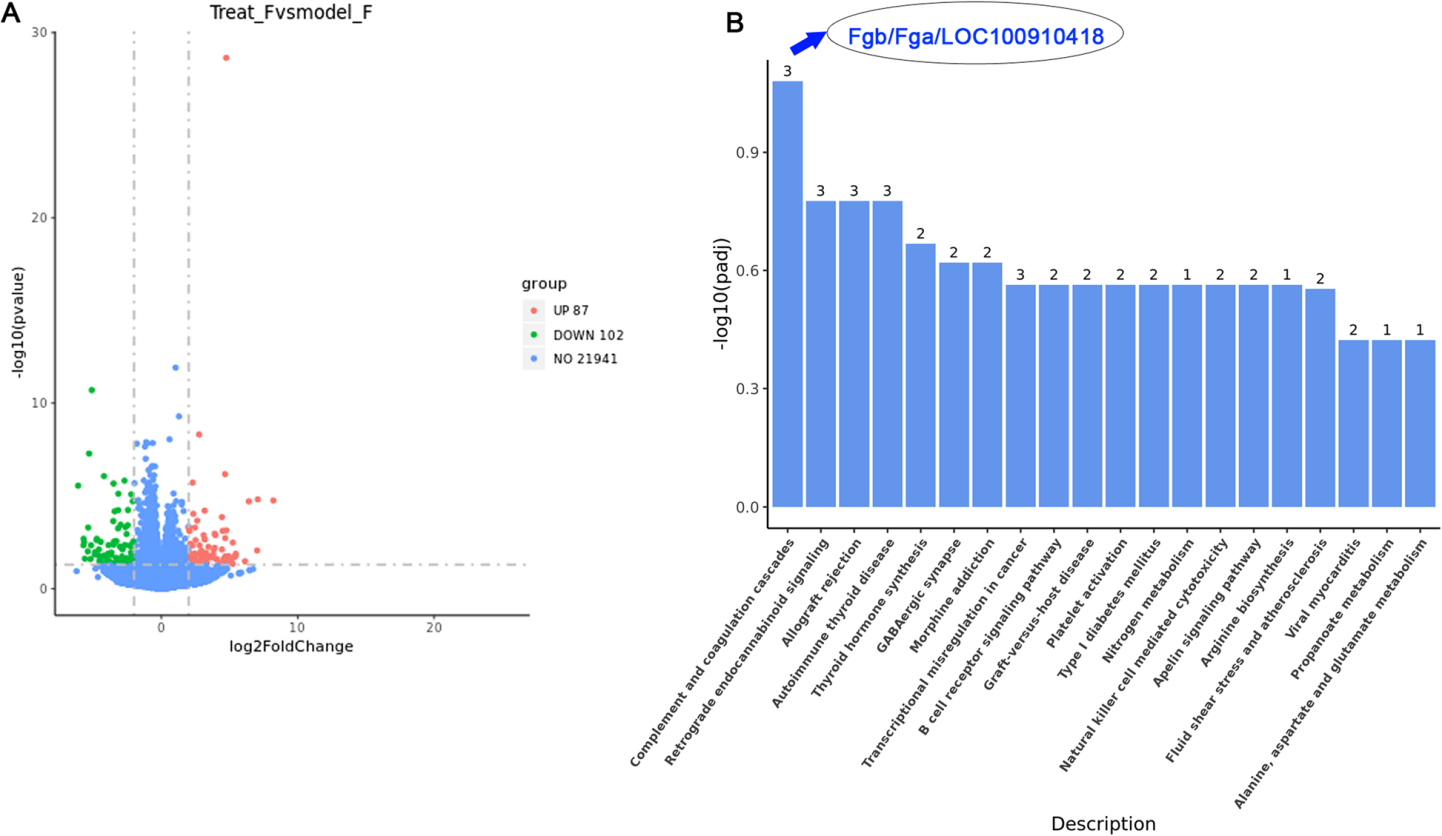
Effect of VD3 on transcriptome differential genes in hypoxia-induced rat model. Thirty SD rats were randomly divided into the normoxia group, hypoxia group, and vitamin D3 (VD3, 1,25-(OH)2-D3) group with 10 rats in each group. **A** Pearson Correlation test was performed for statistical analysis, and the results were presented in volcano plot. Volcano plot of differential gene expression analysis. **B** KEGG pathways enrichment statistical analysis was performed by KOBAS software. KEGG derived Bar graph of the significantly enriched pathways

**Fig. 3:**
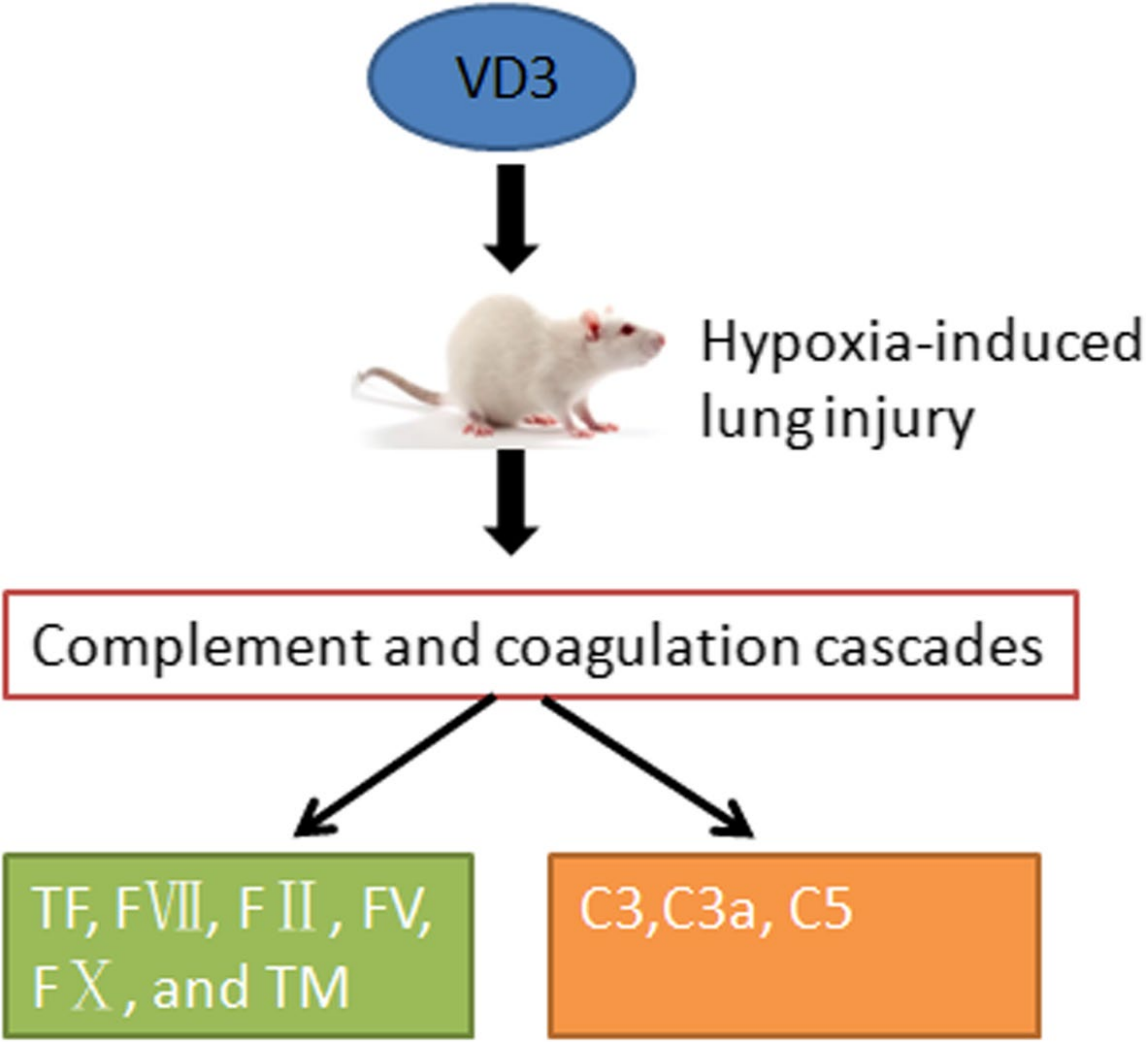
Effect of VD3 on complement and coagulation cascades in a hypoxia-induced rat model. VD3 regulated coagulation cascades are TF, FVII, FII, FV, FX, and TM, and the C3/C5 pathway in the downstream complement cascades

Transcriptome analysis revealed the therapeutic mechanism of VD3 in high-altitude pulmonary edema. As shown in Fig. 2A, 87 mRNAs were significantly upregulated and 102 mRNAs were significantly downregulated after VD3 treatment compared to those in the hypoxic group. These differentially enriched gene pathways were analyzed using KEGG, and the results are shown in Fig. 2B. The complement and coagulation cascade pathways were the most significant pathways for differential gene enrichment, and the genes enriched in these pathways were Fgb/Fga/LOC100910418. Therefore, the therapeutic effect of VD3 on high-altitude pulmonary edema may be related to the regulatory role of the complement and coagulation cascade pathway in Fgb/Fga gene enrichment. The factors involved in the regulation of coagulation cascades are TF, FVII, FII, FV, FX, and TM, and they participate in the regulation of the C3/C5 pathway in the downstream complement cascades (Fig. 3 and Supplementary Fig. 1).

### VD3 inhibited inflammation and lung permeability disruption caused by a hypoxic environment

**Fig. 4:**
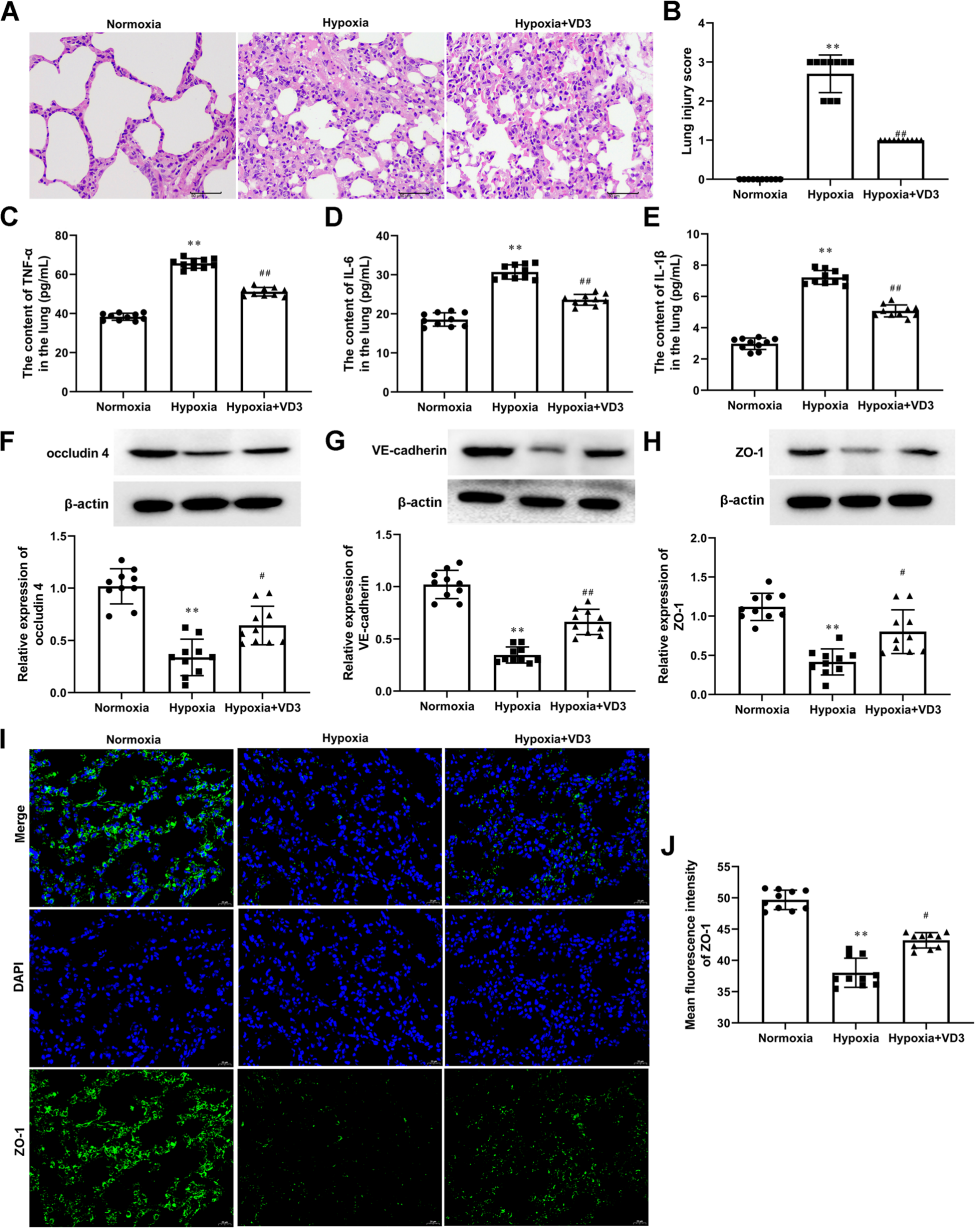
VD3 inhibited inflammation and lung permeability disruption caused by hypoxic environment. Thirty SD rats were randomly divided into the normoxia group, hypoxia group, and vitamin D3 (VD3, 1,25-(OH)2-D3) group with 10 rats in each group. **A** and **B** Lung injury score assessed based on evaluation of H&E stain. **C**-**E** The concentrations of TNF-α, IL-6, and IL-1β in lung tissues were determined by ELISA. **F**–**H** The expression of occludin 4, VE-cadherin, and ZO-1 in lung tissues were evaluated by Western blot. β-actin served as a loading control. **I** and **J** IF stain for ZO-1 (magnification, × 400). Means and standard deviations (SD) were used to represent the data. Statistical analyses (two group comparisons) were performed using Students *t*-test. ^*^ *P* < 0.05 vs Normoxia, ^**^ *P* < 0.01 vs Normoxia, ^#^ *P* < 0.05 vs Hypoxia, ^##^ *P* < 0.01 vs Hypoxia

As displayed in Fig. 4A and B, alveolar hemorrhage, inflammatory cell infiltration, and alveolar wall thickening were observed in the hypoxia group (0.00 ± 0.00 vs 2.70 ± 0.48), which were inhibited by VD3 treatment (2.70 ± 0.48 vs 1.00 ± 0.00). Meanwhile, hypoxia increased the expression of TNF-α (38.36 ± 1.88 vs 65.57 ± 2.63), IL-6 (18.56 ± 1.82 vs 30.72 ± 1.94), and IL-1β (2.98 ± 0.39 vs 7.22 ± 0.47, Fig. 4C-E). Compared with the hypoxia group, VD3 decreased the expression of TNF-α (65.57 ± 2.63 vs 51.16 ± 2.35), IL-6 (30.72 ± 1.94 vs 23.60 ± 1.48), and IL-1β (7.22 ± 0.47 vs 5.08 ± 0.41, Fig. 4C-E). Western blot and IF showed that hypoxia downregulated the junction proteins ZO-1 (Western blot, 1.12 ± 0.19 vs 0.42 ± 0.18; IF, 49.69 ± 1.65 vs 38.03 ± 2.47), occludin 4 (1.02 ± 0.18 vs 0.34 ± 0.19), and vascular endothelial cadherin (VE-cadherin; 1.00 ± 0.14 vs 0.35 ± 0.08) compared with the normoxia group (Fig. 4F-J). However, after the administration of VD3, the expression of ZO-1 (Western blot, 0.42 ± 0.18 vs 0.80 ± 0.30; IF, 38.03 ± 2.47 vs 43.21 ± 1.31), occludin 4 (0.34 ± 0.19 vs 0.64 ± 0.20), and VE-cadherin (0.35 ± 0.08 vs 0.66 ± 0.13) increased (Fig. 4F-J).

### VD3 suppressed Fga and Fgb expression in the lung of hypoxia-induced rats

**Fig. 5:**
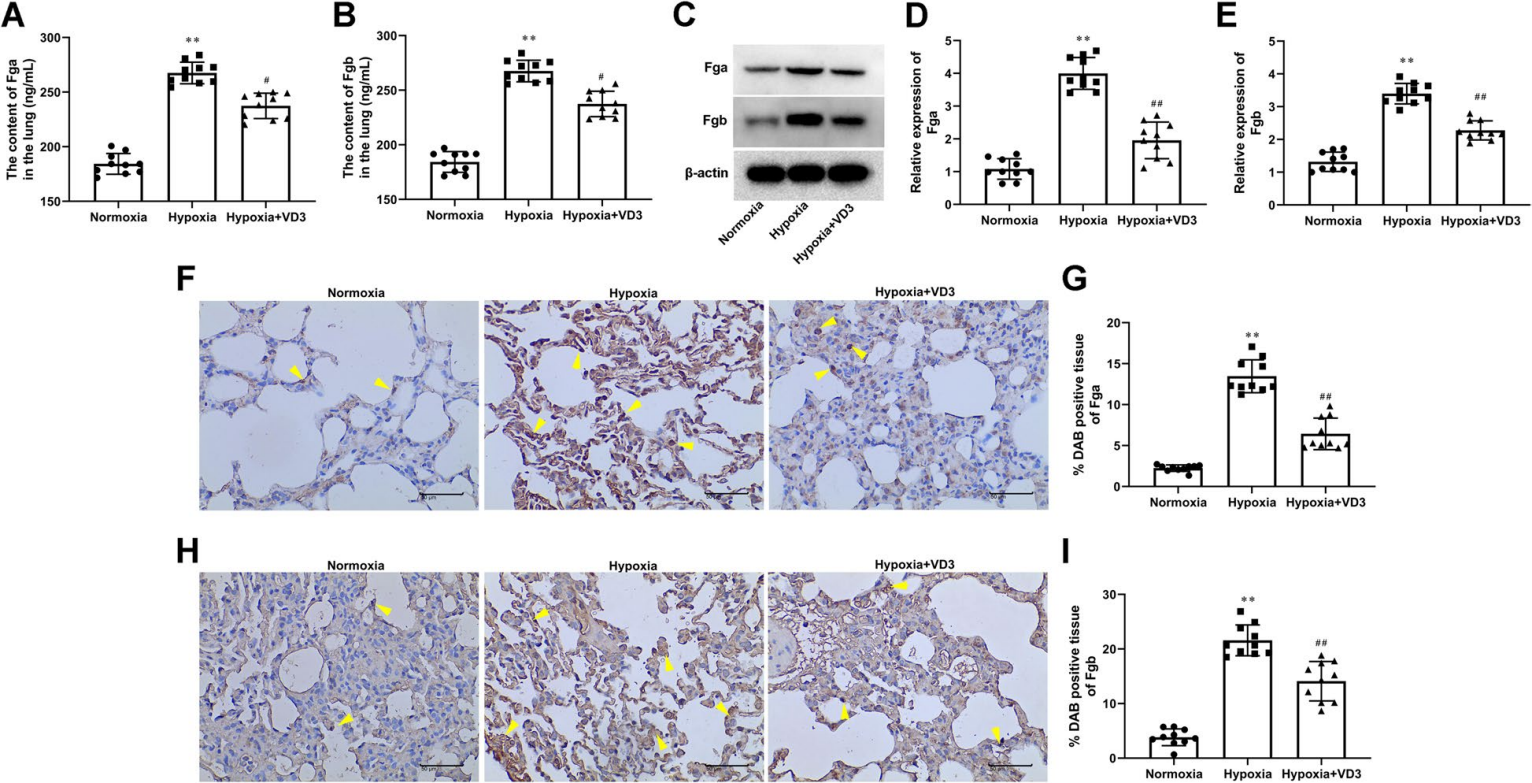
VD3 suppressed Fga and Fgb expression in lung of hypoxia-induced rats. Thirty SD rats were randomly divided into the normoxia group, hypoxia group, and vitamin D3 (VD3, 1,25-(OH)2-D3) group with 10 rats in each group. **A** and **B** ELISA analysis of Fga and Fgb in lung tissues. **C**-**E** Expression of Fga and Fgb was detected by Western blot. Load control: β-actin. **F** and **G** IHC detection of Fga (magnification, × 400). **H** and **I** Fgb detection was performed by IHC stain (magnification, × 400). Arrows indicate cells that are positively expressed. Means and standard deviations (SD) were used to represent the data. Statistical analyses (two group comparisons) were performed using Students *t*-test. ^**^ *P* < 0.01 vs Normoxia, ^#^ *P* < 0.05 vs Hypoxia, ^##^ *P* < 0.01 vs Hypoxia

Fga/Fgb genes encode fibrinogens, which may play an important role in hypoxia-induced inflammation [34]. The ELISA, western blot, and IHC results suggested that hypoxia enhanced Fga (ELISA, 186.41 ± 10.17 vs 266.92 ± 10.43; the blot, 1.08 ± 0.34 vs 4.00 ± 0.51; IHC, 2.25 ± 0.42 vs 13.47 ± 2.13) and Fgb (ELISA, 184.32 ± 10.03 vs 267.58 ± 10.51; Western blot, 1.32 ± 0.32 vs 3.40 ± 0.33; IHC, 3.84 ± 1.63 vs 21.58 ± 2.99) expression in lung tissues. Furthermore, the increased Fga (ELISA, 266.92 ± 10.43 vs 235.41 ± 11.25; Western blot, 4.00 ± 0.51 vs 1.96 ± 0.59; IHC, 13.47 ± 2.13 vs 6.43 ± 2.05) and Fgb (ELISA, 267.58 ± 10.51 vs 237.44 ± 12.38; Western blot, 3.40 ± 0.33 vs 2.27 ± 0.31; IHC, 21.58 ± 2.99 vs 14.10 ± 3.84) expression induced by hypoxia were reversed by VD3 treatment (Fig. 5A-I).

### VD3 weakened coagulation cascade in the lung and liver of hypoxia-induced rats

**Fig. 6:**
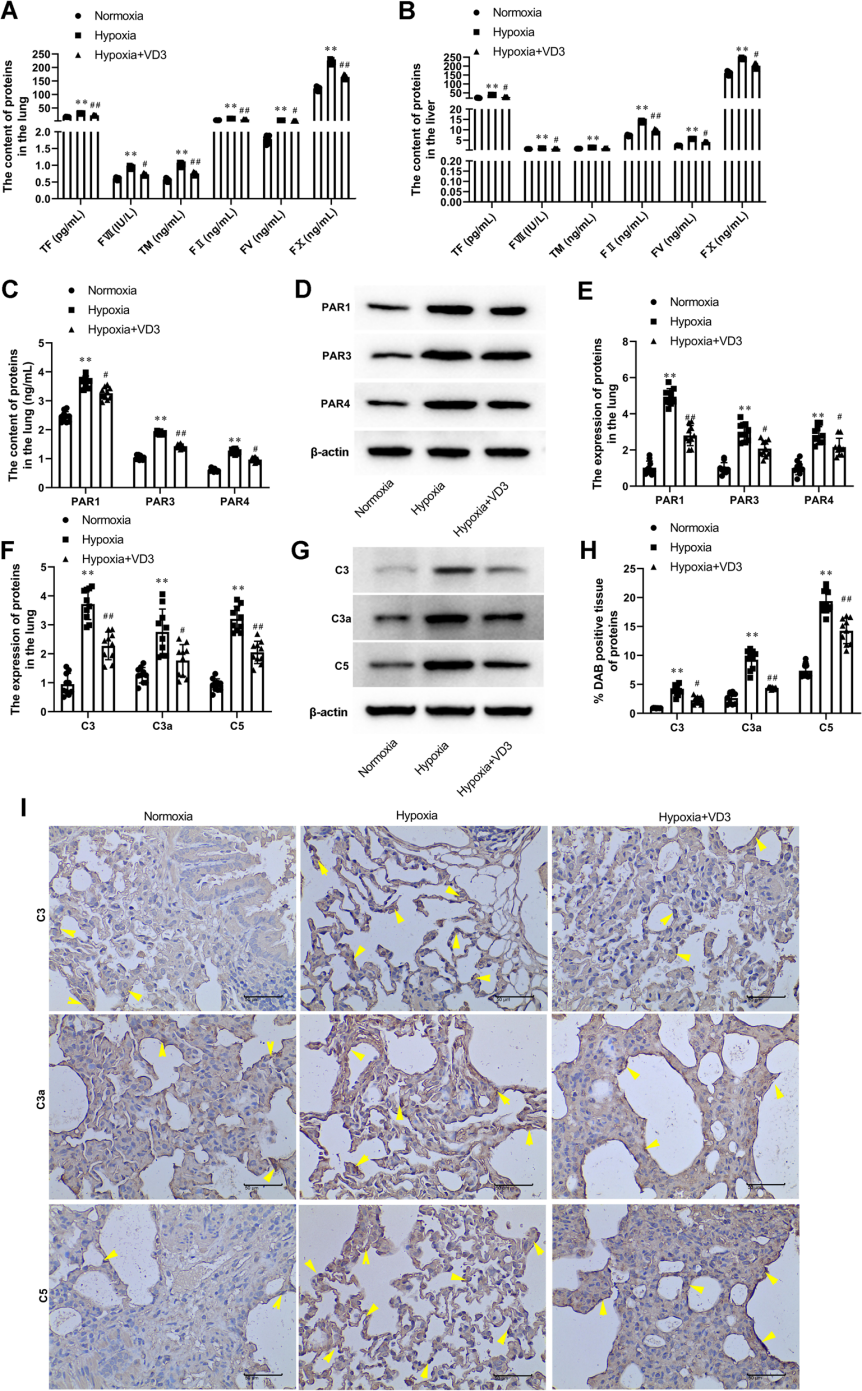
VD3 weakened coagulation cascade in lung and liver of hypoxia-induced rats. Thirty SD rats were randomly divided into the normoxia group, hypoxia group, and vitamin D3 (VD3, 1,25-(OH)2-D3) group with 10 rats in each group. **A** and **B** The protein levels of TF, FVII, FII, FV, FX, and TM in lung and liver was tested by ELISA. **C** The contents of PAR1, PAR3, and PAR4 in lung was detected by ELISA. **D** and **E** The expression of PAR1, PAR3, and PAR4 in lung was analyzed by Western blot. Load control: β-actin. **F** and **G** The expression of C3, C3a, and C5 in lung were assayed by Western blot analysis. Load control: β-actin. **H** and **I** IHC detection of C3, C3a, and C5 in lung (magnification, × 400). Arrows indicate cells that are positively expressed. Means and standard deviations (SD) were used to represent the data. Statistical analyses (two group comparisons) were performed using Students *t*-test. ^**^ *P* < 0.01 vs Normoxia, ^#^ *P* < 0.05 vs Hypoxia, ^##^ *P* < 0.01 vs Hypoxia

ELISA assay revealed that hypoxia exposure resulted in a significant increase in TF (lung, 17.17 ± 0.87 vs 30.25 ± 1.33; liver, 22.70 ± 1.12 vs 39.33 ± 1.27), FVII (lung, 0.60 ± 0.04 vs 0.93 ± 0.04; liver, 0.78 ± 0.02 vs 1.11 ± 0.05), TM (lung, 0.56 ± 0.05 vs 1.02 ± 0.05; liver, 0.94 ± 0.04 vs 1.46 ± 0.04), FII (lung, 5.06 ± 0.36 vs 10.95 ± 0.26; liver, 7.25 ± 0.35 vs 13.95 ± 0.41), FV (lung, 1.78 ± 0.12 vs 4.39 ± 0.07; liver, 2.44 ± 0.08 vs 5.63 ± 0.15), and FX (lung, 121.60 ± 7.40 vs 220.09 ± 7.98; liver, 159.45 ± 10.37 vs 250.87 ± 10.82) in lung and liver of high-altitude pulmonary edema rats, which was blocked by VD3 treatment ((TF (lung, 30.25 ± 1.33 vs 22.91 ± 1.22; liver, 39.33 ± 1.27 vs 27.43 ± 1.15), FVII (lung, 0.93 ± 0.04 vs 0.73 ± 0.03; liver, 1.11 ± 0.05 vs 0.93 ± 0.04), TM (lung, 1.02 ± 0.05 vs 0.75 ± 0.04; liver, 1.46 ± 0.04 vs 1.10 ± 0.02), FII (lung, 10.95 ± 0.26 vs 7.80 ± 0.45; liver, 13.95 ± 0.41 vs 9.51 ± 0.64), FV (lung, 4.39 ± 0.07 vs 2.63 ± 0.13; liver, 5.63 ± 0.15 vs 4.14 ± 0.15), and FX (lung, 220.09 ± 7.98 vs 164.95 ± 7.34; liver, 250.87 ± 10.82 vs 202.60 ± 12.80); Fig. 6A and B)). Meanwhile, hypoxic preconditioning promoted the levels of PAR1 (ELISA, 2.45 ± 0.19 vs 3.70 ± 0.24; Western blot, 1.00 ± 0.39 vs 5.00 ± 0.47), PAR3 (ELISA, 1.04 ± 0.08 vs 1.89 ± 0.06; Western blot, 1.00 ± 0.33 vs 3.01 ± 0.52), and PAR4 (ELISA, 0.61 ± 0.06 vs 1.26 ± 0.08; Western blot, 1.00 ± 0.38 vs 2.83 ± 0.50; Fig. 6C-E). VD3 treatment also resulted in a significant decrease in PAR1 (ELISA, 3.70 ± 0.24 vs 3.26 ± 0.16; Western blot, 5.00 ± 0.47 vs 2.80 ± 0.59), PAR3 (ELISA, 1.89 ± 0.06 vs 1.43 ± 0.08; Western blot, 3.01 ± 0.52 vs 2.08 ± 0.47), and PAR4 (ELISA, 1.26 ± 0.08 vs 0.98 ± 0.07; Western blot, 2.83 ± 0.50 vs 2.16 ± 0.52) levels in the lung compared with the hypoxia group (Fig. 6C-E). Additionally, Western blot and IHC staining showed that the expression of C3 (Western blot, 0.95 ± 0.40 vs 3.72 ± 0.57; IHC, 0.90 ± 0.05 vs 3.96 ± 0.90), C3a (Western blot, 1.26 ± 0.28 vs 2.76 ± 0.84; IHC, 2.64 ± 1.02 vs 9.27 ± 1.77), C5 (Western blot, 0.90 ± 0.23 vs 3.21 ± 0.47; IHC, 7.37 ± 1.23 vs 19.39 ± 2.14) was increased in the hypoxia group (Fig. 6F-I). The aforementioned changes in proteins were inhibited by VD3 administration (C3 (Western blot, 3.72 ± 0.57 vs 2.27 ± 0.51; IHC, 3.96 ± 0.90 vs 2.27 ± 0.68); C3a (Western blot, 2.76 ± 0.84 vs 1.77 ± 0.58; IHC, 9.27 ± 1.77 vs 4.39 ± 0.20), C5 (Western blot, 3.21 ± 0.47 vs 2.05 ± 0.41; IHC, 19.39 ± 2.14 vs 14.27 ± 2.34); Fig. 6F-I).

### VD3 attenuated autophagy in the lungs of hypoxia-induced rats

**Fig. 7:**
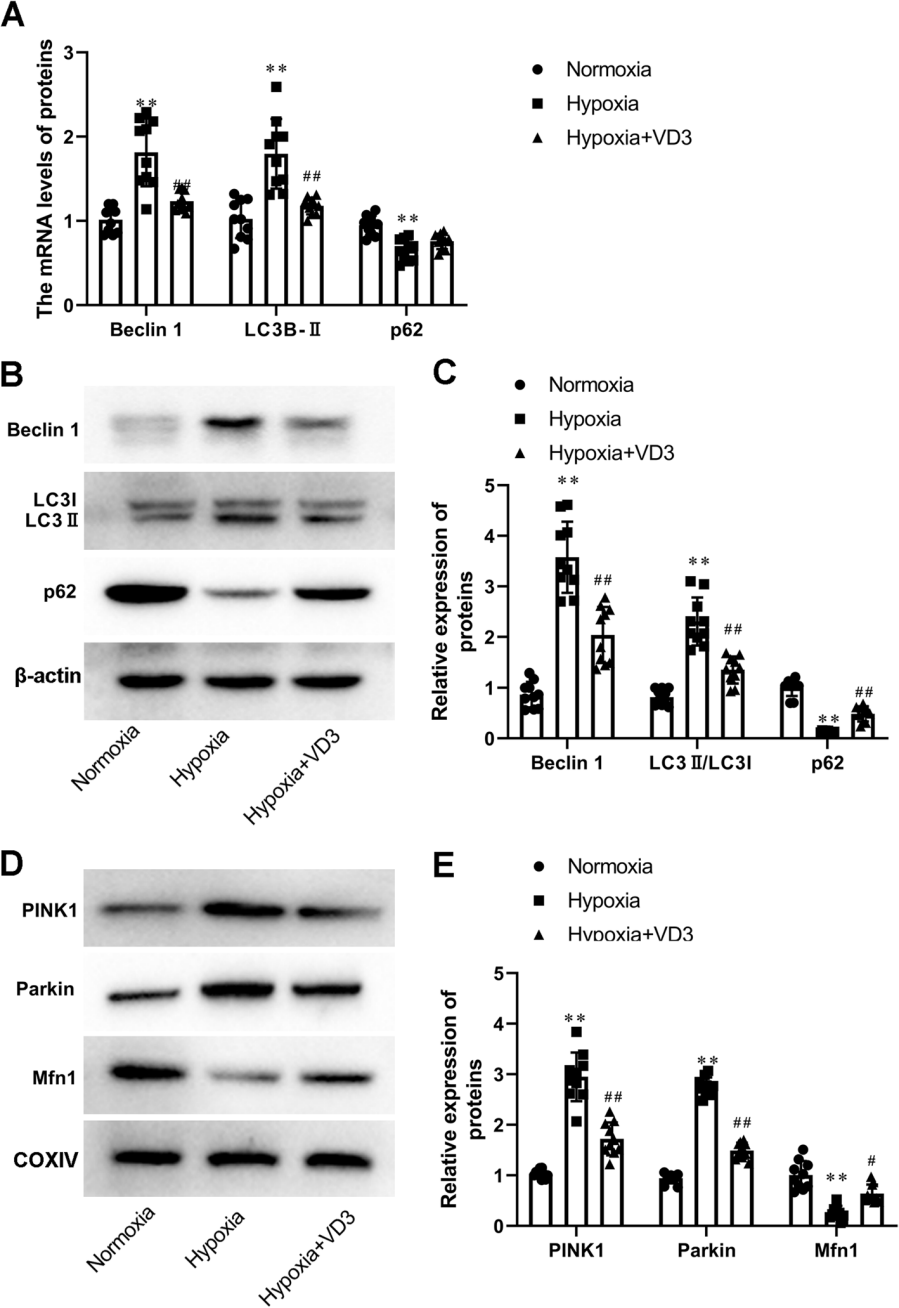
VD3 attenuated autophagy in lung of hypoxia-induced rats. Thirty SD rats were randomly divided into the normoxia group, hypoxia group, and vitamin D3 (VD3, 1,25-(OH)2-D3) group with 10 rats in each group. **A** mRNA levels of LC3B-II, p62, and Beclin-1were determined through RT-qPCR. **B** and **C** The expression of LC3 I, LC3 II, p62, and Beclin-1 in lung were assessed using Western blot analysis. Load control: β-actin. **D** and **E** PINK1, Parkin, and Mfn1 protein expression were measured by Western blot analysis. COXIV, mitochondria loading control. Means and standard deviations (SD) were used to represent the data. Statistical analyses (two group comparisons) were performed using Students *t*-test. ^**^ *P* < 0.01 vs Normoxia, ^#^ *P* < 0.05 vs Hypoxia, ^##^ *P* < 0.01 vs Hypoxia

Next, we investigated the effect of VD3 on autophagy in rats with high-altitude pulmonary edema. LC3B I, LC3B II, p62, and Beclin-1 are central autophagy-related proteins involved in autophagic flux. The expression of LC3B, Beclin-1, and p62 was analyzed using RT-qPCR and Western blotting. As shown in Fig. 7A-C, LC3B (RT-qPCR, 1.02 ± 0.24 vs 1.80 ± 0.45; Western blot, 0.82 ± 0.17 vs 2.31 ± 0.51) and Beclin 1 (RT-qPCR, 1.01 ± 0.17 vs 1.81 ± 0.43; Western blot, 0.87 ± 0.27 vs 3.60 ± 0.75) expression in the hypoxia group was significantly induced compared with that in the normoxia group, whereas LC3B (RT-qPCR, 1.80 ± 0.45 vs 1.18 ± 0.11; Western blot, 2.31 ± 0.51 vs 1.35 ± 0.28) and Beclin-1 (RT-qPCR, 1.81 ± 0.43 vs 1.23 ± 0.12; Western blot, 3.60 ± 0.75 vs 2.04 ± 0.59) content in the VD3 group was significantly decreased compared with that in the hypoxia group. In contrast, decreased levels of p62 (RT-qPCR, 1.00 ± 0.12 vs 0.66 ± 0.14; Western blot, 1.01 ± 0.18 vs 0.13 ± 0.03) were observed in the hypoxia group, which was enhanced by VD3 treatment (RT-qPCR, 0.66 ± 0.14 vs 0.76 ± 0.10; Western blot, 0.13 ± 0.03 vs 0.49 ± 0.16; Fig. 7A-C). In addition, PINK1, Parkin, and Mfn1 are key molecules involved in mitophagy regulation. Figure 7D and E showed with PINK1(1.03 ± 0.09 vs 2.95 ± 0.51) and Parkin (0.94 ± 0.18 vs 2.76 ± 0.19) the normoxia group, the hypoxia group showed a significant increase. VD3 treatment decreased the expression of PINK1 (2.95 ± 0.51 vs 1.72 ± 0.35) and Parkin (2.76 ± 0.19 vs 0.49 ± 0.17) in the lung of high-altitude pulmonary edema rats (Fig. 7D and E). And Mfn1 levels showed opposite changes (1.01 ± 0.30 vs 0.26 ± 0.15, 0.26 ± 0.15 vs 0.64 ± 0.19; Fig. 7D and E).

### VD3 improved type II alveolar epithelial cell damage and inflammation induced by hypoxia

**Fig. 8:**
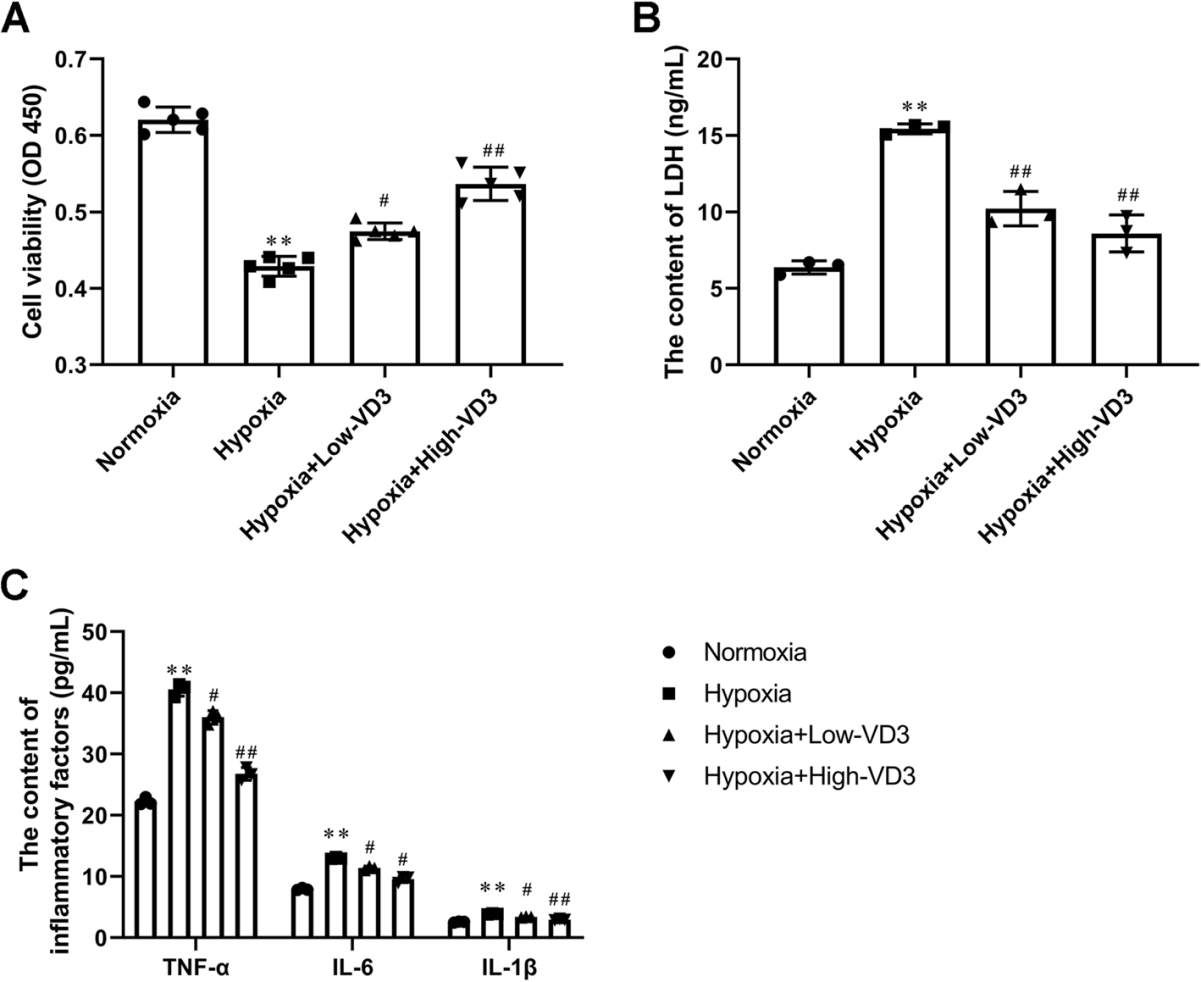
VD3 improved type II alveolar epithelial cell damage and inflammation induced by hypoxia. The cells were divided into four groups: normoxia group, hypoxia group, hypoxia + low-concentration VD3 group, and hypoxia + high-concentration VD3 group. **A** CCK-8 assay of type II alveolar epithelial cell proliferation. **B** LDH activity was determined using a commercial LDH assay kit. **C** Expression levels of cellular supernatant TNF-α, IL-6 and IL-1β were determined by ELISA. Means and standard deviations (SD) were used to represent the data. Statistical analyses (two group comparisons) were performed using Students *t*-test. A one-way ANOVA with Tukey post hoc test of means was used for multiple group comparisons. ^**^ *P* < 0.01 vs Normoxia, ^#^ *P* < 0.05 vs Hypoxia, ^##^ *P* < 0.01 vs Hypoxia

The protective effect of VD3 against hypoxia-induced alveolar epithelial cell injury was investigated in vitro. The CCK-8 assay revealed that hypoxia decreased cell proliferation (0.62 ± 0.12 vs 0.43 ± 0.01), which was rescued by a low (0.43 ± 0.01 vs 0.47 ± 0.01) and high (0.43 ± 0.01 vs 0.54 ± 0.02) dose of VD3 (Fig. 8A). To further assess cell injury, LDH leakage was tested using the LDH assay. LDH release (6.38 ± 0.43 vs 15.44 ± 0.32) was increased in the co-culture system of alveolar epithelial cells and hepatocytes subjected to hypoxic injury, whereas the addition of VD3 low (15.44 ± 0.32 vs 10.22 ± 1.13) and high (15.44 ± 0.32 vs 8.60 ± 1.21) dose to type II alveolar epithelial cells exposed to hypoxic injury led to decreased LDH release (Fig. 8B). ELISA results suggested that hypoxia-induced TNF-α (22.25 ± 0.63 vs 40.57 ± 1.10), IL-6 (7.94 ± 0.18 vs 13.01 ± 0.17), and IL-1β (2.59 ± 0.10 vs 3.89 ± 0.19) expression, which was reversed by the low (TNF-α, 40.57 ± 1.10 vs 36.00 ± 1.10; IL-6, 13.01 ± 0.17 vs 11.37 ± 0.30; IL-1β, 3.89 ± 0.19 vs 3.39 ± 0.10) and high (TNF-α, 40.57 ± 1.10 vs 26.77 ± 1.03; IL-6, 13.01 ± 0.17 vs 9.54 ± 0.54; IL-1β, 3.89 ± 0.19 vs 2.96 ± 0.13) dose of VD3 (Fig. 8C).

### VD3 inhibited complement and coagulation cascade in hypoxia-induced co-culture system of alveolar epithelial cells and hepatocytes

**Fig. 9:**
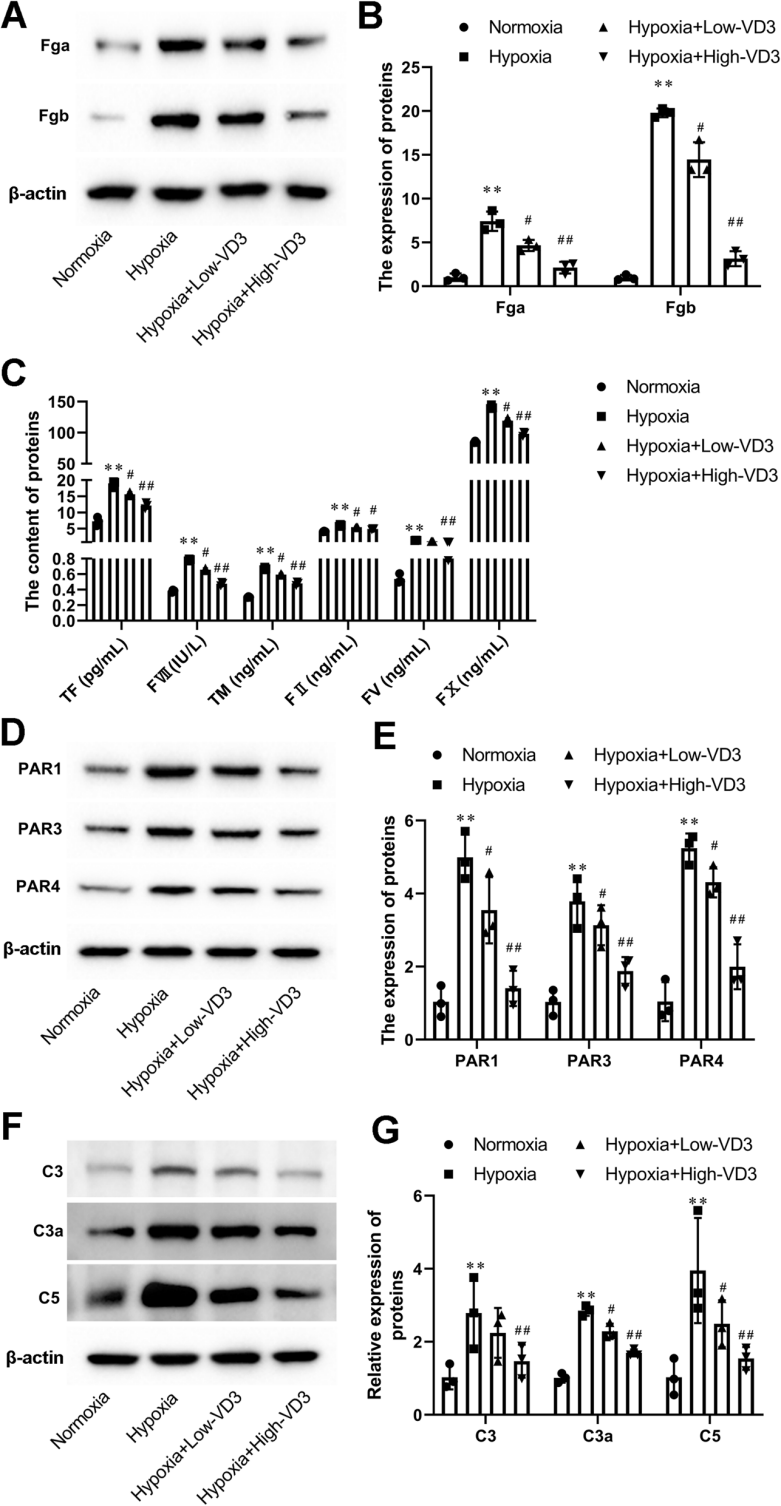
VD3 inhibited complement and coagulation cascade in hypoxia-induced co-culture system of alveolar epithelial cells and hepatocytes. **A** and **B** Expression of Fga and Fgb was detected by Western blot. Load control: β-actin. **C** The protein levels of TF, FVII, FII, FV, FX, and TM in type II alveolar epithelial cells were tested by ELISA. **D**-**G** The expression of PAR1, PAR3, PAR4, C3, C3a, and C5 in type II alveolar epithelial cells was analyzed by Western blot. Load control: β-actin. Means and standard deviations (SD) were used to represent the data. Statistical analyses (two group comparisons) were performed using Students *t*-test. A one-way ANOVA with Tukey post hoc test of means was used for multiple group comparisons. ^**^ *P* < 0.01 vs Normoxia, ^#^ *P* < 0.05 vs Hypoxia, ^##^ *P* < 0.01 vs Hypoxia

As shown in Fig. 9A and B, Fga (1.04 ± 0.43 vs 7.42 ± 1.11) and Fgb (0.99 ± 0.31 vs 19.81 ± 0.50) expression markedly increased under hypoxic conditions compared with normoxic conditions. Interestingly, treatment of hypoxic alveolar epithelial cells with a low (Fga, 7.42 ± 1.11 vs 4.67 ± 0.64; Fgb, 19.81 ± 0.50 vs 14.46 ± 1.98) and high dose (Fga, 7.42 ± 1.11 vs 2.13 ± 0.66; Fgb, 19.81 ± 0.50 vs 3.15 ± 0.85) of VD3 prevented upregulation of Fga and Fgb expression (Fig. 9A and B). VD3 low (TF, 18.83 ± 1.04 vs 15.64 ± 0.67; FVII, 0.79 ± 0.01 vs 0.66 ± 0.02; TM, 0.67 ± 0.02 vs 0.59 ± 0.01; FII, 6.03 ± 0.30 vs 5.43 ± 0.13; FV, 1.40 ± 0.07 vs 1.08 ± 0.06 and FX, 141.33 ± 3.20 vs 119.41 ± 1.06) and high dose (TF, 18.83 ± 1.04 vs 12.11 ± 0.93; FVII, 0.79 ± 0.01 vs 0.48 ± 0.02; TM, 0.67 ± 0.02 vs 0.48 ± 0.02; FII, 6.03 ± 0.30 vs 4.89 ± 0.17; FV, 1.40 ± 0.07 vs 0.81 ± 0.05 and FX, 141.33 ± 3.20 vs 98.14 ± 3.51) treatment markedly blunted the hypoxia-induced increase in protein levels of TF, FVII, TM, FII, FV, and FX (Fig. 9C). Furthermore, significant increases in PAR1 (1.04 ± 0.43 vs 4.99 ± 0.66), PAR3 (1.03 ± 0.36 vs 3.79 ± 0.69), and PAR4 (1.04 ± 0.54 vs 5.24 ± 0.41) were observed in hypoxia-induced alveolar epithelial cells, which were decreased by low (PAR1, 4.99 ± 0.66 vs 3.55 ± 0.91; PAR3, 3.79 ± 0.69 vs 3.13 ± 0.55; PAR4, 5.24 ± 0.41 vs 4.31 ± 0.41) and high (PAR1, 4.99 ± 0.66 vs 1.41 ± 0.48; PAR3, 3.79 ± 0.69 vs 1.88 ± 0.39; PAR4, 5.24 ± 0.41 vs 2.00 ± 0.61) dose of VD3 (Fig. 9D and E). In Fig. 9F and G, we show that the increases in C3 (1.03 ± 0.32 vs 2.80 ± 0.98), C3a (1.01 ± 0.13 vs 2.85 ± 0.14), and C5 (1.03 ± 0.50 vs 3.95 ± 1.44) expression observed in hypoxic condition was completely abolished by VD3 low (C3, 2.80 ± 0.98 vs 2.24 ± 0.68; C3a, 2.85 ± 0.14 vs 2.29 ± 0.21; C5, 3.95 ± 1.44 vs 2.49 ± 0.64) and high dose (C3, 2.80 ± 0.98 vs 1.47 ± 0.45; C3a, 2.85 ± 0.14 vs 1.70 ± 0.10; C5, 3.95 ± 1.44 vs 1.54 ± 0.31) treatment.

### VD3 eliminated autophagy in hypoxia-induced co-culture system of alveolar epithelial cells and hepatocytes

**Fig. 10:**
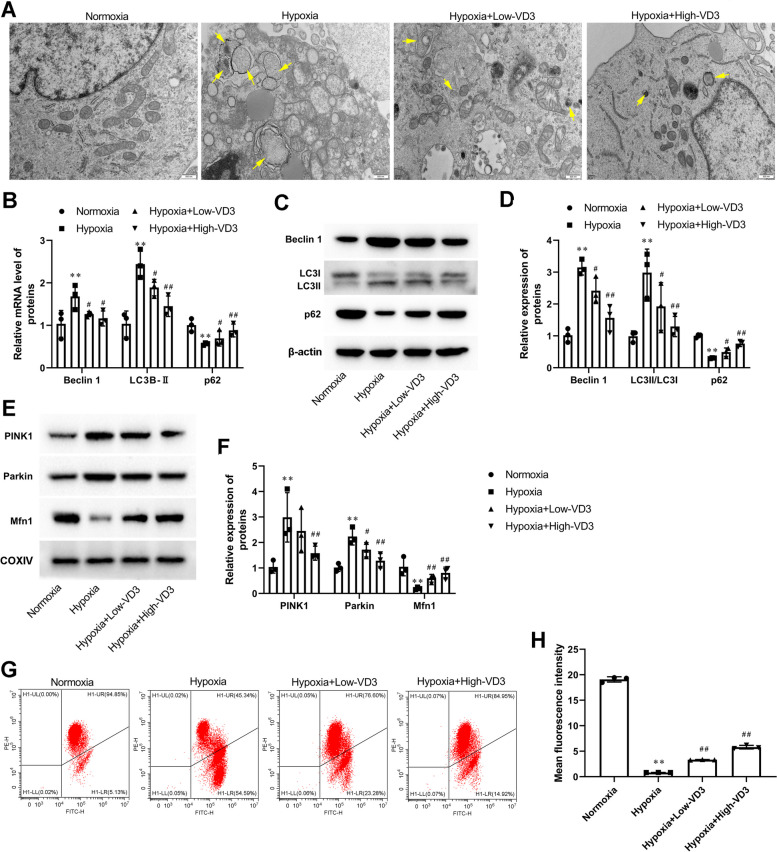
VD3 eliminated autophagy in hypoxia-induced co-culture system of alveolar epithelial cells and hepatocytes. **A** The images of TEM showed that autophagosome accumulation in hypoxia-induced alveolar epithelial cells (magnification, × 20,000). **B** mRNA levels of LC3B-II, p62, and Beclin-1 were determined through RT-qPCR. **C** and **D** The expression of LC3 I, LC3 II, p62, and Beclin-1 in lung were assessed using Western blot analysis. Load control: β-actin. **E** and **F** PINK1, Parkin, and Mfn1 protein expression were measured by Western blot analysis. COXIV, mitochondria loading control. **G** and **H** Flow cytometric analysis of mitochondrial membrane potential by JC-1 in hypoxia-induced alveolar epithelial cells. Means and standard deviations (SD) were used to represent the data. Statistical analyses (two group comparisons) were performed using Students *t*-test. A one-way ANOVA with Tukey post hoc test of means was used for multiple group comparisons. ^**^ *P* < 0.01 vs Normoxia, ^#^ *P* < 0.05 vs Hypoxia, ^##^ *P* < 0.01 vs Hypoxia

In this study, TEM was used to observe the ultrastructure of hypoxia-induced alveolar epithelial cells (Fig. 10A). Compared to normal cells, numerous autophagosomes consisting of double membranes were observed in hypoxia-induced alveolar epithelial cells (Fig. 10A). VD3 treatment inhibited autophagosome formation (Fig. 10A). mRNA (LC3B II, 1.04 ± 0.30 vs 2.44 ± 0.33; Beclin 1, 1.04 ± 0.32 vs 1.68 ± 0.29) and protein (LC3B II, 0.99 ± 0.17 vs 2.98 ± 0.74; Beclin 1, 1.02 ± 0.21 vs 3.15 ± 0.24) expression of LC3B II and Beclin 1 significantly increased upon hypoxic exposure, and this effect was stopped when hypoxic alveolar epithelial cells were treated with low (mRNA: LC3B II, 2.44 ± 0.33 vs 1.89 ± 0.19; mRNA: Beclin 1, 1.68 ± 0.29 vs 1.27 ± 0.06; protein: LC3B II, 2.98 ± 0.74 vs 1.92 ± 0.75; protein: Beclin 1, 3.15 ± 0.24 vs 2.42 ± 0.40) and high (mRNA: LC3B II, 2.44 ± 0.33 vs 1.45 ± 0.24; mRNA: Beclin 1, 1.68 ± 0.29 vs 1.16 ± 0.18; protein: LC3B II, 2.98 ± 0.74 vs 1.29 ± 0.7532; protein: Beclin 1, 3.15 ± 0.24 vs 1.57 ± 0.42) dose VD3 (Fig. 10B-D). In contrast, decreased p62 levels (mRNA:1.00 ± 0.16 vs 0.56 ± 0.03; protein:1.00 ± 0.05 vs 0.30 ± 0.02) were observed in hypoxia-induced alveolar epithelial cells, which were blocked by the low (mRNA:0.56 ± 0.03 vs 0.69 ± 0.18; protein:0.30 ± 0.02 vs 0.49 ± 0.13) and high (mRNA:0.56 ± 0.03 vs 0.88 ± 0.15; protein:0.30 ± 0.02 vs 0.76 ± 0.09) dose of VD3 (Fig. 10B-D). More importantly, the upregulation of PINK1 and Parkin protein expression was completely suppressed (PINK1, 1.04 ± 0.24 vs 2.99 ± 0.97; Parkin, 1.02 ± 0.15 vs 2.23 ± 0.33) when hypoxic alveolar epithelial cells were incubated with low (PINK1, 2.99 ± 0.97 vs 2.45 ± 0.85; Parkin, 2.23 ± 0.33 vs 1.72 ± 0.28) and high (PINK1, 2.99 ± 0.97 vs 1.58 ± 0.27; Parkin, 2.23 ± 0.33 vs 1.29 ± 0.29) dose VD3 (Fig. 10E-F). Meanwhile, the expression level of Mfn1 was decreased (1.05 ± 0.35 vs 0.20 ± 0.05) under the hypoxic condition but was restored by low (0.20 ± 0.05 vs 0.60 ± 0.15) and high (0.20 ± 0.05 vs 0.80 ± 0.23) dose VD3 treatment (Fig. 10E-F). Finally, mitochondrial membrane potential (Δψm) was decreased (19.09 ± 0.52 vs 0.83 ± 0.01) upon hypoxia but was significantly increased by low (0.83 ± 0.01 vs 3.33 ± 0.05) and high (0.83 ± 0.01 vs 5.80 ± 0.35) dose VD3 treatment (Fig. 10G and H).

### Autophagy activation reversed the protective effect of VD3 on alveolar epithelial cells against hypoxic injury

**Fig. 11:**
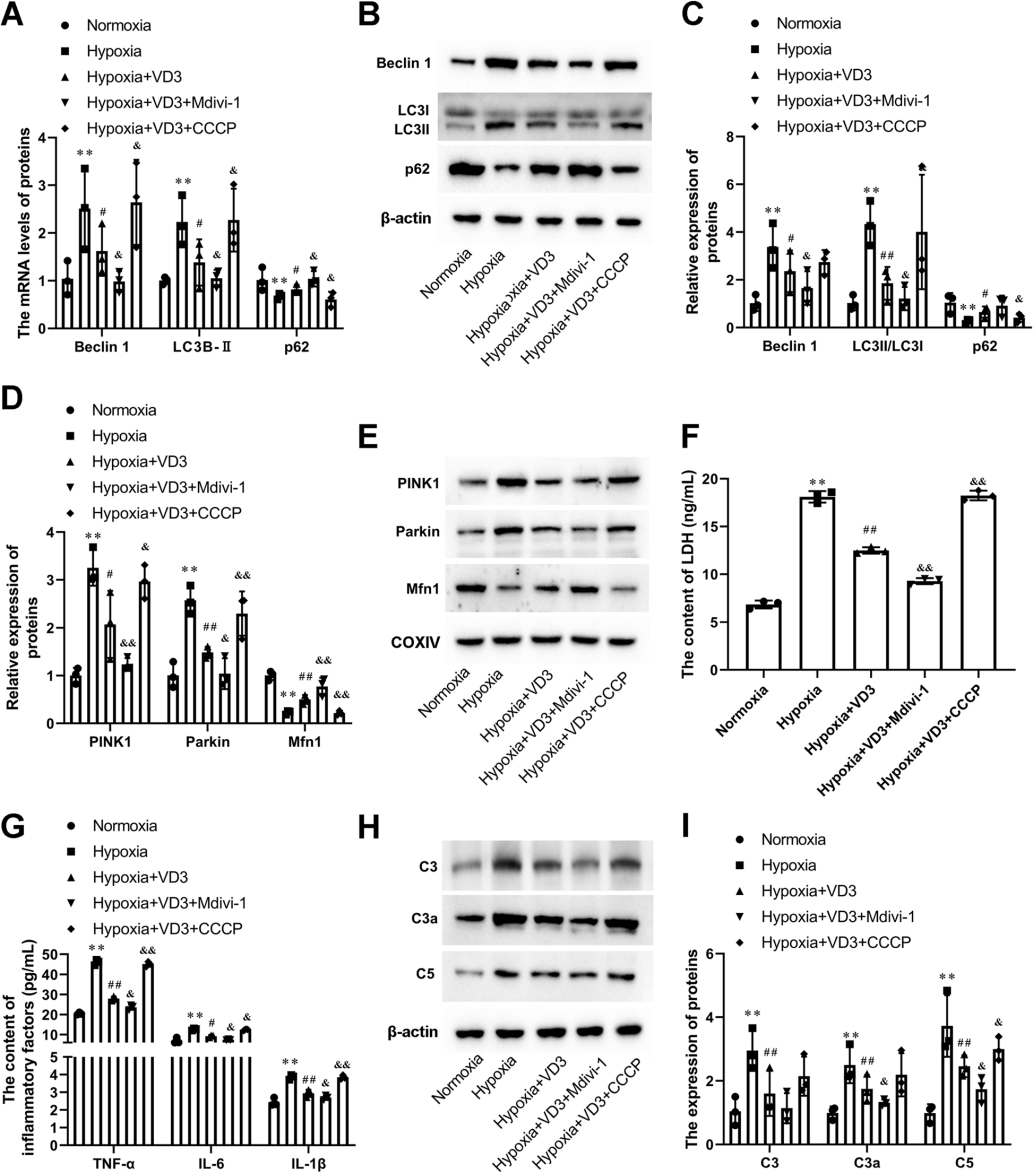
Autophagy activation reversed the protective effect of VD3 on alveolar epithelial cells against hypoxic injury. The cells were divided into 5 groups: normoxia group, hypoxia group, hypoxia + VD3 group, hypoxia + VD3 + mitochondrial autophagy inhibitor (5 μM Mdivi-1) group and hypoxia + VD3 + mitochondrial autophagy agonist (50 μM CCCP) group. **A** mRNA levels of LC3B-II, p62, and Beclin-1 were determined through RT-qPCR. **B** and **C** The expression of LC3 I, LC3 II, p62, and Beclin-1 in lung were assessed using Western blot analysis. Load control: β-actin. **D** and **E** PINK1, Parkin, and Mfn1 protein expression were measured by Western blot analysis. COXIV, mitochondria loading control. **F** LDH activity was measured at 490 nm using the LDH cytotoxicity kit. **G** Expression levels of cellular supernatant TNF-α, IL-6 and IL-1β were determined by ELISA. **H** and **I** The expression of C3, C3a, and C5 in type II alveolar epithelial cells was analyzed by Western blot. Load control: β-actin. Means and standard deviations (SD) were used to represent the data. Statistical analyses (two group comparisons) were performed using Student *t*-test. A one-way ANOVA with Tukey post hoc test of means was used for multiple group comparisons. ^**^ *P* < 0.01 vs Normoxia, ^#^ *P* < 0.05 vs Hypoxia, ^##^ *P* < 0.01 vs Hypoxia, ^&^*P* < 0.05 vs Hypoxia + VD3, ^&&^*P* < 0.01 vs Hypoxia + VD3

Finally, Mdivi-1or CCCP were used to inhibit or promote mitophagy in hypoxia-induced alveolar epithelial cells. As shown in Fig. 11A-C, treatment of alveolar epithelial cells with VD3 significantly reduced (mRNA: LC3B II, 2.23 ± 0.52 vs 1.38 ± 0.48; mRNA: Beclin 1, 2.51 ± 0.85 vs 1.62 ± 0.52; protein: LC3B II, 4.34 ± 0.94 vs 1.86 ± 0.69; protein: Beclin 1, 3.38 ± 0.94 vs 2.36 ± 0.87) the hypoxia-induced increase in LC3B II and Beclin 1 protein expression, which was further inhibited by Mdivi-1 (mRNA: LC3B II, 1.38 ± 0.48 vs 1.05 ± 0.18; mRNA: Beclin 1, 1.62 ± 0.52 vs 0.98 ± 0.22; protein: LC3B II, 1.86 ± 0.69 vs 1.22 ± 0.49; protein: Beclin 1, 2.36 ± 0.87 vs 1.66 ± 0.71) and was reversed by CCCP (mRNA: LC3B II, 1.38 ± 0.48 vs 2.27 ± 0.66; mRNA: Beclin 1, 1.62 ± 0.52 vs 2.64 ± 0.89; protein: LC3B II, 1.86 ± 0.69 vs 4.01 ± 2.40; protein: Beclin 1, 2.36 ± 0.87 vs 2.75 ± 0.50). The expression levels of P62 (mRNA: 0.68 ± 0.06 vs 0.82 ± 0.10, 0.82 ± 0.10 vs 1.04 ± 0.16, and 0.82 ± 0.10 vs 0.61 ± 0.16; protein: 0.29 ± 0.09 vs 0.65 ± 0.23, 0.65 ± 0.23 vs 0.92 ± 0.43, and 0.65 ± 0.23 vs 0.41 ± 0.18) showed opposite results (Fig. 11A-C). In addition, we observed PINK1 (1.00 ± 0.17 vs 3.25 ± 0.37) and Parkin (1.00 ± 0.27 vs 2.57 ± 0.29) in hypoxic alveolar epithelial cells (Fig. 11D and E). The accumulation of PINK1 (3.25 ± 0.37 vs 2.07 ± 0.70) and Parkin (2.57 ± 0.29 vs 1.48 ± 0.12) were blocked by treatment with VD3 (Fig. 11D and E). Mdivi-1 was further eliminated (PINK1, 2.07 ± 0.70 vs 1.23 ± 0.14; Parkin, 1.48 ± 0.12 vs 1.04 ± 0.32) and CCCP strengthened (PINK1, 2.07 ± 0.70 vs 2.97 ± 0.35; Parkin, 1.48 ± 0.12 vs 2.30 ± 0.46) PINK1 and Parkin expression compared with the VD3-treated group (Fig. 11D and E). The expression levels of Mfn1 showed opposite results (0.23 ± 0.04 vs 0.50 ± 0.09, 0.50 ± 0.09 vs 0.77 ± 0.19, and 0.50 ± 0.09 vs 0.22 ± 0.05; Fig. 11D and E). LDH levels in the supernatants were detected using an LDH kit. Mdivi-1 further promoted (2.65 ± 0.95 vs 6.18 ± 0.17) the inhibitory effect of VD3 on LDH release, which was abolished by CCCP (2.65 ± 0.95 vs 0.64 ± 0.02, Fig. 11F). Interestingly, the addition of VD3 in the culture medium significantly protected alveolar epithelial cells from hypoxia-induced inflammation (TNF-α, 46.06 ± 1.36 vs 27.92 ± 1.02; IL-6, 12.65 ± 0.54 vs 9.23 ± 0.54; IL-1β, 3.86 ± 0.13 vs 2.94 ± 0.21), which was also reinforced by Mdivi-1 (TNF-α, 27.92 ± 1.0 vs 23.58 ± 1.24; IL-6, 9.23 ± 0.54 vs 7.43 ± 0.25; IL-1β, 2.94 ± 0.21 vs 2.69 ± 0.130) and was destructed by CCCP (TNF-α, 27.92 ± 1.0 vs 45.09 ± 1.20; IL-6, 9.23 ± 0.54 vs 12.45 ± 0.39; IL-1β, 2.94 ± 0.21 vs 3.85 ± 0.12, Fig. 11G). Moreover, we observed that treatment with VD3 prevented C3 (2.95 ± 0.64 vs 1.60 ± 0.71), C3a (2.50 ± 0.57 vs 1.75 ± 0.43), and C5 (3.73 ± 0.97 vs 2.46 ± 0.34) under hypoxic conditions (Fig. 11H and I). In addition, compared with VD3-treated cells, Mdivi-1 prevented C3 (1.60 ± 0.71 vs 1.14 ± 0.47), C3a (1.75 ± 0.43 vs 1.31 ± 0.10), and C5 (2.46 ± 0.34 vs 1.74 ± 0.41) accumulation, as well as CCCP promoted C3 (1.60 ± 0.71 vs 2.15 ± 0.62), C3a (1.75 ± 0.43 vs 2.19 ± 0.68), and C5 (1.75 ± 0.43 vs 2.99 ± 0.37) accumulation, although this effect was partial (Fig. 11H and I).

## Discussion

In our study, we observed increases in blood K^+^ and Na^+^ concentrations under hypoxic conditions. VD3 treatment significantly reduced K^+^ and Na^+^ concentrations and ultimately maintained electrolyte homeostasis. Patients with lung injury caused by hyperbaric hypoxia may also present with generalized edema and pleural-pericardial effusion, and the resulting heart failure may be the cause of elevated K^+^ and Na^+^ levels [35, 36]. Similarly, a previous study showed that Compared with controls, patients with lung injury caused by hyperbaric hypoxia had significantly higher serum Na + and K^+^ [37]. In addition, when the altitude increased to 3200 m, the subjects' blood potassium levels increased significantly [38]. Mechanistically, the hypoxia-induced closure of K^+^ conductance in alveolar epithelium results in fluid clearance, thereby promoting fluid retention and contributing to the development of pulmonary edema [39]. A further investigation into alveolar epithelial cells has confirmed that hypoxia leads to a down-regulation of the expression and epithelial Na^+^ channels (ENaC) and Na, K-ATPase, thereby diminishing salt and water clearance [40]. Moreover, in response to alveolar hypoxia, a mitochondrial sensor dynamically changes reactive oxygen species and redox couples in pulmonary artery smooth muscle cells (PASMC), inhibiting potassium channels, and ultimately inducing depolarization of PASMC [41]. Hypoxia inhibited several potassium channels (voltage-gated and TASK), leading to membrane depolarization. The inhibition of potassium channels results in high cytosolic levels of potassium. These lead to the inhibition of apoptosis and an increase in cellular proliferation [42].

Previously published data have demonstrated in cases of high-altitude pulmonary edema, there is an increase in the infiltration of inflammatory cells, specifically macrophages and neutrophils, as well as elevated levels of cytokines such as IL-6, TNF-α, and IL-1β [43]. Rats exposed to acute hypobaric hypoxia and exhibiting signs of high-altitude pulmonary edema displayed an upregulation of NF-κB levels in the nuclear fraction [44]. This increase in NF-κB activity regulates the production of inflammatory molecules, including IL-1, IL-6, and TNF-α, within the lung tissue under hypoxic conditions [44]. Furthermore, this study highlights an elevation in the levels of cell adhesion molecules ICAM-1 and VCAM-1 [44]. Furthermore, a separate study conducted on rats experiencing high-altitude pulmonary edema (HAPE) as a result of acute hypobaric hypoxia (9142 m for 5 h) also demonstrated elevated levels of proinflammatory molecules, including TNF-α, monocyte chemoattractant protein-1 (MCP-1), INF-γ, IL-6, and TNF-β, within the bronchoalveolar lavage. Additionally, an increase in NF-κB levels was observed in lung nuclear extracts [45]. Our data suggest that VD3 inhibited the protein levels of inflammatory biomarkers, such as IL-6, TNF-α, and IL-1β, caused by a hypoxic environment in vivo and in vitro.

The disruption of the integrity of the lung vascular endothelial barrier leads to an increase in permeability, resulting in pulmonary edema [46]. VE-Cadherin is specifically expressed in endothelial cells and is considered to be a structurally and functionally critical part of adherence junctions [47]. A previous study indicated that hypoxia selectively disrupts microvascular endothelial tight junction complexes in the lung through the permeability-inducing factors VEGF and VE-cadherin [48]. In addition, increased capillary pressure was found to induce pulmonary permeability edema by disrupting endothelial adhesion junctions, including activation of the calcium-dependent protease calpain and degradation of adhesion junction proteins VE-Cadherin, β-catenin, and p120-Catenin [49]. Tight junction (TJ) proteins occludin and ZO-1 are reported to be critical for regulating pulmonary vascular permeability. Ulinastatin ameliorates pulmonary edema by upregulating the expression of ZO-1 and occludin, thereby reducing pulmonary permeability and stimulating alveolar fluid clearance [50]. The TJ protein (ZO-1, JAM-C, claudin-4, occludin) expression in the lungs of the HAPE model group has been restored to a normal high level upon quercetin pre-treatment [51]. The results of the present study demonstrate that VD3 treatment effectively ameliorated hypoxia-aggravated pulmonary barrier injury, as evidenced by the significant increase in the expression of ZO-1, occludin, and VE-Cadherin.

The clotting factors, anticoagulation factors, and fibrinolytic system of the coagulation pathway are in a dynamic balance and work together to maintain [52, 53]. Once this balance is disturbed, abnormal bleeding or thrombosis occurs. Fibrinogen (Fg) is an important factor in the coagulation pathway and consists of an alpha chain (Fga), beta chain (Fgb), and gamma chain (Fgg) [54]. Fibrinolytic inhibition prevents an increase in lung vascular permeability after pulmonary thromboembolism [55]. Furthermore, fibrinogen augmented mean arterial pressure and reduced histopathological injury and lung permeability by inhibiting MMP-9-mediated syndecan-1 cleavage in obese mice [56]. Tissue factor (TF) is a crucial component of the coagulation pathway. Upon tissue injury, exposure of plasma to TF expressed on non-vascular cells or activated endothelial cells leads to the formation of the TF-factor VIIa (FVIIa) complex [57, 58]. Subsequent catalysis of the initial activation of factor X (FX) to FXa is facilitated by the TF-FVIIa complex. Subsequently, the enzymatic conversion of prothrombin into thrombin (TM) is facilitated by the collaborative action of FXa and activated factor V (FVa) [59]. Sustained coagulation is achieved through the catalytic action of thrombin, synthesized via the initial TF-FVIIa-FXa complex, which activates FXI, FIX, FVIII, and FX [60, 61]. The study showed that uncontrolled activation of the coagulation cascade contributes to acute and chronic lung diseases [58]. FX expression of FX was locally increased in human and murine fibrotic lung tissues [62]. FX inhibitors attenuated bleomycin-induced pulmonary fibrosis in mice [62]. Significantly increased levels of TF-enriched neutrophil extracellular traps (NETs) have been observed in patients with acute respiratory distress syndrome (ARDS) patients and mice. The blockade of NETs in ARDS mice alleviated disease progression, indicating a reduced lung wet/dry ratio and PaO_2_ levels [63]. In this study, we found that VD3 prevented the upregulation of Fga and Fgb expression in the lungs of rats with high-altitude pulmonary edema. Meanwhile, VD3 treatment markedly blunted the hypoxia-induced increase in the protein levels of TF, FVII, FII, FV, FX, and TM. Meanwhile, in the co-culture system of alveolar epithelial cells and hepatocytes, VD3 treatment blunted the hypoxia-induced increase in the protein levels of TF, FVII, FII, FV, FX, and TM.

Protease-activated receptors (PARs) are a superfamily of G protein-coupled receptors that mediate transmembrane signaling and regulate cellular functions, including four members: PAR1, PAR2, PAR3, and PAR4. They are mainly distributed in the airways, intestines, skin, and other tissues where inflammatory responses are likely to occur [64, 65]. The biological effects of PARs include inducing a coagulation response, releasing inflammatory factors to regulate the local inflammatory response, increasing vascular exudation and neutrophil chemotaxis, regulating vascular tone, and promoting cell division and proliferation [64, 66]. Current research indicates that PARs play important roles in embryonic development, atherosclerosis, vascular stenosis, and the physiological and pathological processes of tumors [64, 67, 68]. Studies have shown that the activity of PARs after activation is mainly characterized by the high expression of inflammatory mediators such as IL-6, IL-8, cyclooxygenase, prostacyclin, angiogenesis, and regulation of vascular barrier function [69–71]. PAR1-mediated enhancement of alpha (v) beta 6-dependent TGF-β activation could be one mechanism by which activation of the coagulation cascade contributes to the development of acute lung injury [72]. In an ischemia/reperfusion-induced acute lung injury (ALI) model, the specific PAR-1 antagonist SCH530348 decreased lung edema and neutrophil infiltration, attenuated thrombin production, reduced inflammatory factors, including cytokine-induced neutrophil chemoattractant-1, IL-6, and TNF-α, mitigated lung cell apoptosis, and downregulated phosphoinositide 3-kinase (PI3K), nuclear factor-κB (NF-κB), and mitogen-activated protein kinase (MAPK) pathways [73]. PAR1, PAR3, and PAR4-induced epithelial-mesenchymal transition (EMT) have been suggested to be a possible mechanism underlying the expanded (myo) fibroblast pool in lung fibrosis [74]. γ-Tocotrienol (γ-TE) inhibits IL-13/STAT6-activated eotaxin secretion in human lung epithelial A549 cells via upregulation of PAR4 expression and enhancement of atypical protein kinase C (aPKC)-PAR4 complex formation [75]. Our experiments demonstrated that hypoxia enhanced the expression of PAR1, PAR3, and PAR4, both in vivo and in vitro. VD3 treatment decreased PAR1, PAR3, and PAR4 levels in the lungs of rats with high-altitude pulmonary edema and hypoxia-induced alveolar epithelial cells. Additionally, in the co-culture system of alveolar epithelial cells and hepatocytes, VD3 treatment blunted the hypoxia-induced increase in the protein levels of TF, FVII, FII, FV, FX, and TM. Significant increases in PAR1, PAR3, and PAR4 levels were observed in the hypoxia-induced co-culture system of alveolar epithelial cells and hepatocytes, which were decreased by VD3.

The complement system comprises a group of non-specific proteins found in human and vertebrate serum [76]. It is an important component of the intrinsic immune response [77]. The complement system can be activated in three ways: the classic, alternative, and lectin pathways. An activated complement system can eliminate invaders and protect the body. This system can also modify self-cells, such as apoptotic particles and cellular debris, but can also regulate the cell cycle through its ligands and receptors. C3 is a key protein in the complement cascade response and its multiple molecular binding sites are essential for its role [78]. C3 binds to its corresponding receptor protein and plays an important role in the pathological mechanisms of immune defense and inflammation [79]. The binding of C5a and its receptor (C5aR) stimulates the aggregation of neutrophils and macrophages and generates inflammatory mediators during inflammation [80, 81]. In LPS-induced human lung type II pneumocytes (A549), C3 production was increased [82]. C3 was found to play a dominant role in pathogen-specific T-cell and B-cell responses, contributing to the amelioration of *Chlamydia psittaci*-induced pneumonia in mice [83]. Inhibition of the C3a receptor mitigates sepsis-induced acute lung injury by suppressing pyroptosis in pulmonary vascular endothelial cells [84]. Systemic activation of C5a leads to neutrophil (NEUT) activation, sequestration, and adhesion to the pulmonary capillary endothelium, resulting in damage and necrosis of vascular endothelial cells and ALI [85]. In the present study, the expression of C3, C3a, and C5 was increased in the lungs of rats with high-altitude pulmonary edema and a hypoxia-induced co-culture system of alveolar epithelial cells and hepatocytes. The expression of C3, C3a, and C5 were inhibited by VD3.

Autophagy is a highly conserved self-eating process, in which cells degrade long-lived proteins and organelles for recycling. Autophagy is generally activated by conditions of nutrient deprivation but has also been associated with physiological and pathological processes. Autophagy appears to be involved in pulmonary diseases, either beneficially or adversely [86, 87]. Pharmacological inhibition of autophagy with 3-methyladenine (3-MA) significantly improved pulmonary appearance, edema, microvascular dilatation, and arterial oxygenation in rats with hepatopulmonary syndrome (HPS) rats [88]. Autophagy was activated in common bile duct ligation (CBDL) rats and cultured pulmonary microvascular endothelial cells induced by CBDL rat serum [88]. Autophagy activation reduces the sepsis-induced release of inflammatory factors and pulmonary edema through autophagy activation [86]. mTOR in the epithelium promotes LPS-induced ALI through downregulation of autophagy and subsequent activation of NF-κB [89]. A previous study found that sodium tanshinone II sulfonate A (STS) alleviated hypoxia-induced lung edema by promoting apoptosis, inhibiting inflammatory responses, and upregulating autophagy [90]. Additionally, in vivo experiments, LPS-induced severe pulmonary edema was further exacerbated by inhibiting autophagy [91]. An increase in autophagy is associated with the promotion of inflammation, which leads to lung injury [92]. SARS-CoV-2 spike pseudovirions (SCV-2-S) promote autophagic responses [93]. SCV-2-S-induced autophagy triggered inflammatory responses and apoptosis in infected human bronchial epithelial and microvascular endothelial cells [93]. The ethanol extract of the tuber of A. orientale Juzepzuk (EEAO) relieved the pathological features of chronic obstructive pulmonary disease (COPD) by suppressing lung emphysema and autophagy and inducing TNF-α, IL-6, and TGF-β in a mouse model [94]. In this study, we confirmed that the activation of autophagy in a hypoxia-induced co-culture system of alveolar epithelial cells and hepatocytes and the lungs of rats with pulmonary edema was inhibited by VD3, which was further inhibited by the autophagy inhibitor Mdivi-1 and reversed by the autophagy activator CCCP.

## Conclusions

Taken together, hypoxic rats exhibited lung edema and injury, which resulted from activation of the complement and coagulation cascade pathways. However, VD3 treatment attenuated the hypoxia-induced pulmonary edema in rats. VD3 ameliorated hypoxia-induced lung injury by inhibiting the complement and coagulation cascade pathways and autophagy. Similarly, in a hypoxia-induced co-culture system of alveolar epithelial cells and hepatocytes, VD3 inhibited hypoxia-induced lung epithelial cytotoxicity and inflammation by suppressing the complement and coagulation cascade pathways and autophagy. Therefore, VD3 may be a novel therapeutic agent for the treatment of hypoxia-induced lung injury. However, it remains unclear whether autophagy modulates complement and coagulation signaling pathways in the context of high-altitude pulmonary edema injury. Additionally, the mechanisms through which VD3 regulates the complement and coagulation pathways to facilitate lung damage repair remain elusive, which will be further explored in subsequent experiments.

### Supplementary Information

**Additional file 1: Supplementary Figure 1.** Effect of VD3 on KEGG pathway in a hypoxia-induced rat model. The network was built based on KEGG pathway map.

## Data Availability

The raw data required to reproduce the above findings cannot be shared at this time, as the data also form part of an ongoing analysis. If someone wants to request data from this study in the future, please contact the corresponding author Xiaoyan Pu (E-mail: puxiaoyan1975@163.com).

## Abbreviations

VD3: Vitamin D3
SD: Sprague-Dawley
H&E: Hematoxylin-eosin
Fga: Fibrinogen alpha chain
Fgb: Fibrinogen beta chain
PAR1: Protease-activated receptor 1
PAR3: Protease-activated receptor 3
PAR4: Protease-activated receptor 4
C3: Complement
CCCP: Carbonyl cyanide-m-chlorophenylhydrazone
1,25(OH)_2_D_3_: 1α, 25-Dihydroxyvitamin D3
LPS: Lipopolysaccharide
PaCO_2_: Partial pressure of carbon dioxide
PaO_2_: Arterial pamal pressure of oxygen
SaO_2_: Arterial oxygen saturation
Na^+^: Sodium
K^+^: Potassium
Ca^2+^: Calcium
RIN: RNA integrity number
cDNA: Complementary DNA
DEGs: Differentially expressed genes
GO: Gene ontology
KEGG: Kyoto Encyclopedia of Genes and Genomes
ELISA: Enzyme-linked immunosorbent assay
TNF-α: Tumor necrosis factor-alpha
IL-6: Interleukin 6
IL-1β: Interleukin-1beta
TF: Tissue factor
FVII: Coagulation factor VII
FII: Coagulation factor II
FV: Coagulation factor V
FX: Coagulation factor X
TM: Thrombomodulin
LDH: Lactate dehydrogenase
RT-qPCR: Real-time fluorescence quantitative polymerase chain reaction
IHC: Immunohistochemistry
IF: Immunofluorescence
PBS: Phosphate-buffered saline
TEM: Transmission electron microscopy
PAP: Pulmonary arterial pressure
F2: Coagulation Prothrombin
F13: Factor XIII
PARs: Protease-activated receptors
PI3K: Phosphoinositide 3-kinase
NF-κB: Nuclear factor-κB
MAPK: Mitogen-activated protein kinase
EMT: Wpithelial-mesenchymal transition
γ-TE: γ-Tocotrienol
aPKC: Atypical protein kinase C
CBDL: Common bile duct ligation
VE-cadherin: Vascular endothelial cadherin
NEUT: Neutrophil
STS: Sodium tanshinone II sulfonate A
HAPE: High-altitude pulmonary edema
SPF: Specific pathogen free
