# Supplementary material for: Vitamin D3 improved hypoxia-induced lung injury by inhibiting the complement and coagulation cascade and autophagy pathway
Source: BMC Pulm Med. 2024 Jan 2;24:9. doi: 10.1186/s12890-023-02784-y (PMC10759436; doi:10.1186/s12890-023-02784-y)

**Supplementary Figure 1 Effect of VD3 on KEGG pathway in a hypoxia-induced rat model.** The network was built based on KEGG pathway map.


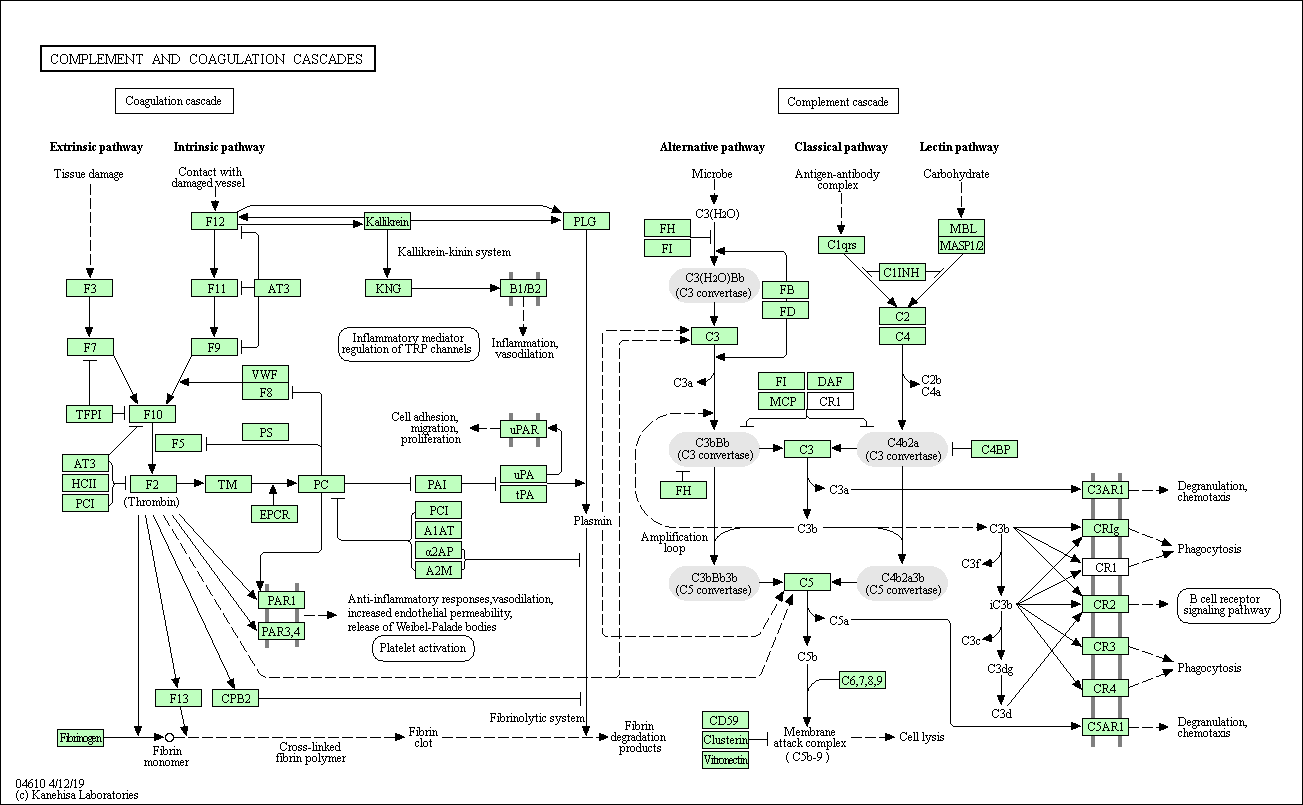

Supplement: Supplementary file 1 — Additional file 1: Supplementary Figure 1. Effect of VD3 on KEGG pathway in a hypoxia-induced rat model. The network was built based on KEGG pathway map. [file 12890_2023_2784_MOESM1_ESM.docx]
